# Integrative Network Pharmacology and Molecular Docking Analysis Reveals the Multitarget Mechanisms of Pterostilbene in Neurodegenerative Diseases

**DOI:** 10.3390/ph19071053

**Published:** 2026-07-08

**Authors:** Natalia Rosiak, Filip Stojceski, Gabriele Maroni, Bartosz Piontek, Judyta Cielecka-Piontek

**Affiliations:** 1 Department of Pharmacognosy and Biomaterials, Faculty of Pharmacy, Poznan University of Medical Sciences, 3 Rokietnicka Street, 60-806 Poznan, Poland; nrosiak@ump.edu.pl; 2 SUPSI, IDSIA-Dalle Molle Institute for Artificial Intelligence (USI-SUPSI), Polo Universitario Lugano-Campus Est, Via la Santa 1, CH-6962 Lugano, Switzerland; filip.stojceski@supsi.ch (F.S.); gabriele.maroni@supsi.ch (G.M.); 3 Poznan Supercoputing and Networking Center, Jana Pawła II 10 Street, 61-139 Poznan, Poland; wasymir@gmail.com

**Keywords:** pterostilbene, network pharmacology, neuroprotection, molecular dynamics, Alzheimer’s disease, Parkinson’s disease, Huntington’s disease, amyotrophic lateral sclerosis

## Abstract

**Background:** Neurodegenerative diseases, including Alzheimer’s disease (AD), Parkinson’s disease (PD), Huntington’s disease (HD), and amyotrophic lateral sclerosis (ALS), differ in etiology but share several convergent pathological mechanisms. Pterostilbene (PTR) is a natural stilbene with reported antioxidant, anti-inflammatory, and neuroprotective properties. This study aimed to prioritize putative PTR-associated targets and biological processes potentially relevant to shared neurodegenerative mechanisms. **Methods:** An integrative in silico workflow combining network pharmacology, protein–protein interaction (PPI) analysis, GO Biological Process (GO BP) enrichment, molecular docking, and molecular dynamics (MD) simulations was applied. GO BP terms were filtered, focused on neurodegeneration- and neuroprotection-related processes, and subjected to REVIGO-based redundancy reduction. Selected targets were further evaluated by docking and 500 ns MD simulations. **Results:** A total of 181, 165, 128, and 109 shared PTR–disease targets were identified for AD, PD, HD, and ALS, respectively. Redundancy-reduced GO BP analysis indicated associations with neuroinflammation, oxidative stress and reactive oxygen species-related responses, programmed cell death, MAPK/ERK- and PI3K/AKT-related signaling, ion and calcium transport, and lipid-, steroid-, or hormone-associated regulation. PPI topology prioritized SRC, ESR1, and HSP90AA1 as recurrent hub–bottleneck proteins, whereas MD-based structural interpretation focused on ESR1 and HSP90AA1. MD analyses indicated stable PTR interactions with both proteins, with ESR1 showing the most favorable predicted interaction profile. **Conclusions:** These findings suggest that PTR may interact with shared neurodegeneration-relevant molecular systems, particularly through ESR1- and HSP90AA1-associated mechanisms. However, the results are exclusively computational and should be interpreted as hypothesis-generating, requiring further experimental validation.

## 1. Introduction

Neurodegenerative diseases, including Alzheimer’s disease (AD), Parkinson’s disease (PD), Huntington’s disease (HD), and amyotrophic lateral sclerosis (ALS), represent one of the greatest challenges of modern medicine due to their complex pathogenesis, limited therapeutic options, and increasing prevalence in the aging population [1,2,3,4]. Although these disorders differ in their primary etiologies, genetic backgrounds, affected neuronal populations, and clinical manifestations, accumulating evidence indicates that they share several convergent pathogenic mechanisms. These include oxidative stress, mitochondrial dysfunction, neuroinflammation, impaired proteostasis and protein quality control, protein aggregation, apoptosis dysregulation, and alterations in stress- and survival-related signaling pathways, including PI3K/Akt and ERK/MAPK signaling [3,5,6,7,8]. Therefore, analyzing AD, PD, HD, and ALS within a common systems-level framework does not imply that these diseases are mechanistically identical. Rather, it allows the identification of shared molecular nodes and biological processes that may contribute to neurodegeneration and may represent broadly relevant targets for multitarget neuroprotective compounds.

This rationale is particularly relevant for natural compounds with pleiotropic biological activity, which may modulate several interconnected pathways rather than a single disease-specific target. Pterostilbene (PTR), a dimethoxylated analog of resveratrol naturally occurring in blueberries, grapes, and Pterocarpus marsupium, has attracted attention due to its antioxidant, anti-inflammatory, anti-apoptotic, and neuroprotective properties [4,9,10]. Compared with resveratrol, PTR exhibits a more favorable pharmacokinetic profile, mainly due to the presence of methoxy groups that increase its lipophilicity, membrane permeability, metabolic stability, and bioavailability [4,9,10]. These properties, together with its ability to cross the blood–brain barrier, make PTR a promising candidate for further investigation in the context of central nervous system (CNS) disorders [4,10].

Recent experimental and review studies further support the neuroprotective potential of PTR. In experimental models of AD, PTR was shown to attenuate Aβ25–35-induced cognitive deficits and neuronal injury through mechanisms involving SIRT1/Nrf2 signaling, antioxidant defense, mitochondrial protection, and inhibition of mitochondria-dependent apoptosis [11]. PTR has also been reported to modulate cognitive impairment, synaptic dysfunction, neuroinflammation, oxidative stress, and neuronal survival in other experimental models relevant to AD and dementia [9,10]. In ischemic brain injury models, PTR reduced neurological dysfunction, oxidative stress, neuroinflammation, and neuronal apoptosis, including mechanisms associated with COX-2 suppression, SIRT1/Nrf2 signaling, and HDAC3/Nrf1-mediated microglial activation [12,13,14]. In addition, PTR has shown neuroprotective effects in models of peripheral neuropathy and MPTP-induced neurotoxicity, further supporting its anti-inflammatory, antioxidant, and anti-apoptotic potential in nervous system disorders [15,16].

Recent reviews have summarized the involvement of PTR in antioxidant and anti-inflammatory responses, lipid metabolism regulation, synaptic function, neurogenesis, apoptosis regulation, mitochondrial homeostasis, and several signaling pathways relevant to CNS disorders, including AMPK/STAT3, Akt, NF-κB, MAPK, and ERK [9,17,18]. Moreover, Socała et al. [4] emphasized that stilbenes are multitarget compounds with promising therapeutic potential in neuropsychiatric and neurological disorders, while also noting that most available evidence still focuses on resveratrol and that data concerning other stilbenes, including PTR, remain comparatively limited. PTR has also been discussed in the context of PD-related mitochondrial retrograde signaling and endogenous stress-response pathways, suggesting that its neuroprotective effects may involve not only direct antioxidant activity but also modulation of intrinsic cellular stress-response mechanisms [19].

However, despite the growing body of evidence supporting the neuroprotective potential of PTR, most available studies remain disease-specific, pathway-focused, or review-based. In particular, previous studies have usually addressed individual experimental models or selected signaling pathways, whereas the shared molecular mechanisms that may underlie PTR activity across different neurodegenerative diseases remain insufficiently characterized. Therefore, the currently available literature reinforces the rationale for a broader systems-level analysis aimed at identifying common PTR-associated molecular targets and pathways across multiple neurodegenerative disorders.

Another important limitation of PTR is its poor aqueous solubility, which may restrict its bioavailability and biological efficacy. Our previous studies on amorphous PTR delivery systems demonstrated that amorphization can substantially improve its solubility and release profile [20], as well as enhance its antioxidant and neuroprotective properties [21]. Nevertheless, improved physicochemical performance alone does not fully explain the molecular basis of PTR-mediated neuroprotection. Therefore, additional target- and pathway-oriented studies are needed to better understand the mechanisms underlying its biological activity.

In this context, the present study employed an integrative in silico workflow combining network pharmacology, protein–protein interaction analysis, functional enrichment, molecular docking, and molecular dynamics simulations to investigate potential PTR-associated targets and pathways across AD, PD, HD, and ALS. The aim of this work was not to provide direct experimental evidence of target engagement, but rather to prioritize candidate molecular targets and generate testable hypotheses for future experimental validation. By integrating disease-related and compound-related target information within a comparative systems-level framework, this study seeks to complement existing experimental evidence and provide a broader view of the potential multitarget mechanisms associated with PTR-mediated neuroprotection.

## 2. Results and Discussion

Neurodegenerative diseases such as AD, PD, HD, and ALS, despite their distinct clinical manifestations, share significant similarities at the molecular level. Common pathogenic mechanisms include impaired protein homeostasis, oxidative stress, mitochondrial dysfunction, and inflammatory processes [19,22,23,24,25]. Identifying key components of these processes is essential for the development of therapies targeting the underlying causes of the diseases rather than merely alleviating symptoms [19].

In recent years, increasing attention has been given to natural compounds with neuroprotective potential, including pterostilbene (PTR), which has demonstrated beneficial biological properties in preclinical studies, such as antioxidant, anti-inflammatory, and neuroprotective effects [4,10,24,26,27,28,29]. However, its precise mechanism of action in the context of neurodegenerative diseases remains not fully understood.

In this study, an in silico approach was employed, including protein–protein interaction (PPI) network analysis, functional enrichment analysis (Gene Ontology—Biological Process, GO BP; Kyoto Encyclopedia of Genes and Genomes, KEGG), molecular docking, and molecular dynamics (MD) simulations, to identify potential molecular targets of PTR and to evaluate its possible interactions with selected proteins.

### 2.1. Selection of Common Targets

To elucidate the potential molecular mechanisms of PTR in neurodegenerative diseases, the identification of common targets between PTR and disease-associated proteins was performed as an initial step. Integrating compound-related targets with disease-specific datasets enables the recognition of shared molecular components that may play a key role in mediating therapeutic effects [30].

**Figure 1:**
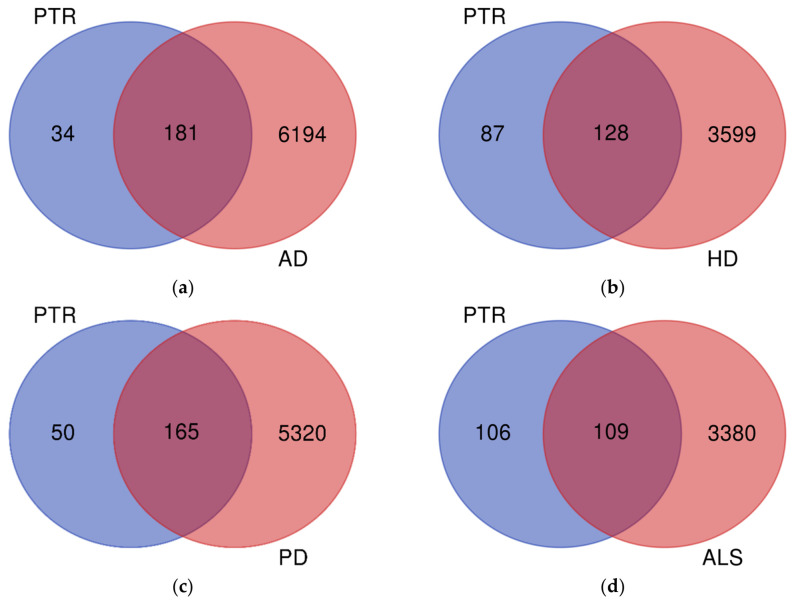
Venn diagram of common targets of pterostilbene (PTR) and (**a**) Alzheimer’s disease (AD), (**b**) Huntington’s disease (HD), (**c**) Parkinson’s disease (PD), (**d**) amyotrophic lateral sclerosis (ALS).

Potential molecular targets of PTR were identified using web-based target prediction tools, including SwissTargetPrediction (n = 97), SuperPred (n = 20), TargetNet (n = 68), and PharmMapper (n = 72) (Supplementary Materials, Table S1). After eliminating duplicate entries, a total of 215 unique PTR targets were obtained (Supplementary Materials, Table S2). Targets associated with Alzheimer’s disease (AD), Huntington’s disease (HD), Parkinson’s disease (PD), and amyotrophic lateral sclerosis (ALS) were extracted from the GeneCards database [31,32]. Venn diagram analysis was performed to identify overlapping targets between PTR and each of the analyzed neurodegenerative diseases (AD, HD, PD, and ALS), and the results are presented in Figure 1.

The Venn diagram of targets related to PTR and AD, HD, PD or ALS showed 181, 128, 165 and 109 shared targets, respectively. Detailed lists of these targets are provided in the Supplementary Materials (Table S3). These collected targets were subsequently used to construct a protein–protein interaction (PPI) giant network.

### 2.2. Construct Protein–Protein Interaction (PPI) Giant Network

The integrated PPI network constructed for targets associated with AD, HD, PD, and ALS enabled the exploration of shared molecular mechanisms that may be associated with neurodegeneration. The resulting giant networks are presented in Supplementary Figures S1–S4. Topological analysis of the individual PPI networks revealed differences in network complexity across the studied diseases, with AD (181 nodes, 363 edges), HD (128 nodes, 255 edges), PD (165 nodes, 334 edges), and ALS (109 nodes, 215 edges). All constructed PPI networks exhibited highly significant enrichment (PPI enrichment *p*-value < 1.0 × 10^−16^), suggesting that the observed number of interactions is greater than expected by chance. This may indicate that the identified proteins are at least partially functionally connected. These observations remain predictive and should be interpreted within the limitations of computational network analysis.

The gene set derived from the constructed PPI network was used as the input for subsequent functional enrichment analysis. This strategy enables the exploration of biological processes and pathways potentially associated with the observed interaction network, although it does not imply direct functional relationships.

### 2.3. Functional Enrichment Analysis

To improve the biological interpretability of the functional enrichment results, GO Biological Process terms were subjected to REVIGO-based semantic clustering [33]. Accordingly, the main interpretation was focused on representative processes related to neurodegeneration and neuroprotection rather than on the complete redundant enrichment output [1]. The complete REVIGO-based semantic clustering results are presented in Supplementary Tables S4–S7 for PTR-AD, PTR-HD, PTR-PD, and PTR-ALS, respectively. For each PTR–disease target set, representative GO BP terms were then ranked by false discovery rate (FDR), and the top 10 terms or representative semantic clusters showing the lowest FDR values were selected for detailed interpretation. The selected top 10 neurodegeneration- and neuroprotection-related GO BP terms are summarized in Supplementary Tables S8–S11 for PTR-AD, PTR-HD, PTR-PD, and PTR-ALS, respectively.

In the obtained results, the representative GO BP terms highlighted several recurring biologically relevant modules in all diseases, including immune-system regulation, learning or memory, MAPK/ERK and PI3K/AKT-related signaling, programmed cell death/apoptotic processes, reactive oxygen species-related stress responses, ion and calcium transport, and lipid/steroid/hormone-associated processes. The recurrence of immune-related terms may reflect the contribution of neuroinflammatory mechanisms, which are increasingly recognized as common contributors to the progression of neurodegenerative diseases [34]. Similarly, the enrichment of ROS- and stress-response-related terms is consistent with the well-established involvement of oxidative imbalance in neuronal dysfunction, mitochondrial damage, and cell death [35,36]. The presence of programmed cell death and apoptotic terms further suggests that PTR-associated targets may be linked to mechanisms regulating neuronal survival. This is relevant because inappropriate activation of apoptotic pathways is considered one of the cellular mechanisms contributing to neuronal loss in neurodegenerative disorders [35]. In addition, MAPK/ERK- and PI3K/AKT-related clusters indicate possible involvement of kinase-mediated signaling networks that may regulate both stress responses and survival pathways. While stress-activated MAPKs may contribute to neuronal injury under pathological conditions, PI3K/AKT and ERK signaling are also frequently discussed in relation to neuronal survival and neuroprotective responses [8]. Ion and calcium transport-related terms may additionally indicate the relevance of neuronal excitability and calcium homeostasis. Dysregulated calcium handling has been implicated in synaptic dysfunction, mitochondrial impairment, excitotoxicity, and neuronal death in several neurodegenerative contexts [37]. Lipid, icosanoid, steroid, and hormone-associated terms may reflect processes linked to membrane homeostasis, inflammatory lipid mediators, and estrogen/steroid-related signaling, all of which have been discussed in the context of neurodegenerative pathology and neuronal resilience [38,39].

### 2.4. Identification of Hub and Bottleneck Nodes in Giant PPI Network

While enrichment analysis provided important insight into the functional landscape of the giant PPI networks, it did not directly prioritize individual nodes according to their relative topological significance. Therefore, to further refine network interpretation, hub and bottleneck node identification was performed. This strategy enables the prioritization of highly connected and centrally positioned proteins that may contribute to network organization and coordination of molecular interactions [40,41,42,43].

**Figure 2:**
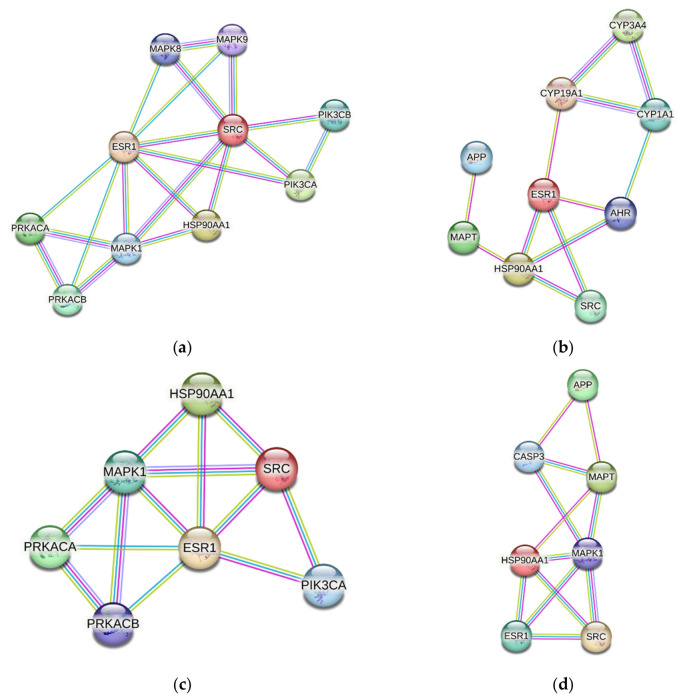

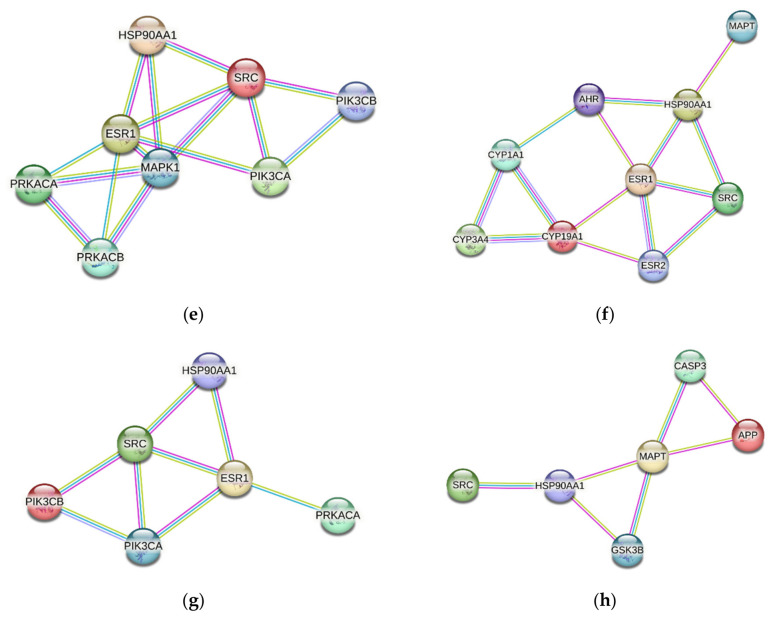
PPI network analysis: (**a**) PTR-AD hub network, (**b**) PTR-AD bottleneck network, (**c**) PTR-HD hub network, (**d**) PTR-HD bottleneck network, (**e**) PTR-PD hub network, (**f**) PTR-PD bottleneck network, (**g**) PTR-ALS hub network, (**h**) PTR-ALS bottleneck network. The figures are available as a high-resolution bitmap (.png format) in the online repository (https://doi.org/10.18150/HNUSRO) in PPI network_results.zip.

To extract biologically relevant information from each giant PPI network, a centrality-based framework was applied to identify putative hub and bottleneck proteins. The resulting subnetworks for AD, HD, PD, and ALS are presented in Figure 2, while detailed rankings of top-prioritized proteins are provided in Tables S12–S15.

Hub nodes were defined as the top 5% of proteins ranked by degree centrality (DC), whereas bottleneck nodes were identified as the top 5% ranked by betweenness centrality (BC). This approach is widely applied in network biology as a practical method for prioritizing candidate proteins with potential structural or regulatory importance within complex interaction systems [40,41,42,43]. Degree centrality was used to identify highly connected proteins that may represent major structural components of the network, whereas betweenness centrality was applied to detect proteins occupying central intermediary positions that could influence communication between distinct network regions or functional modules. Proteins with elevated degree values are often associated with broader interaction capacity, while nodes with high betweenness may indicate critical positions for information flow and network connectivity. However, these topological inferences should be interpreted cautiously, as centrality-based importance reflects network architecture rather than definitive biological function and therefore requires subsequent experimental validation.

The analysis revealed shared hub and bottleneck proteins across the studied networks. For AD, the overlapping proteins (highlighted in green in Tables S12–S15) included C-SRC, ESR1, and HSP90AA1 (Table S12). For HD, common nodes comprised C-SRC, ESR1, HSP90AA1, and MAPK1 (Table S13). For PD, the overlap included C-SRC, ESR1, HSP90AA1, and CYP3A4 (Table S14). In ALS, shared hub–bottleneck proteins included C-SRC and HSP90AA1 (Table S15). These overlapping nodes may indicate proteins occupying central positions in terms of both local connectivity and global network communication. Among these, C-SRC, ESR1, and HSP90AA1 were selected as representative candidates for subsequent molecular docking analysis, based on their consistent occurrence across multiple disease-specific networks.

### 2.5. Molecular Docking

To further assess the potential structural basis of interaction between PTR and the representative proteins prioritized from the shared disease-PTR PPI network analysis, molecular docking was performed for C-SRC, ESR1, and HSP90AA1 and the results are presented in Figure S5.

Although the preceding PPI network analysis identified C-SRC, ESR1, and HSP90AA1 as recurrently prioritized components within the shared molecular landscapes of PTR-associated targets across AD, HD, PD, and ALS, network topology alone does not provide direct structural insight into the feasibility of ligand–target interactions at the molecular level. Therefore, molecular docking was employed to obtain the interaction poses of PTR with SRC, ESR1, and HSP90AA1 that will be used as initial structure for the MD simulations.

This integrative step was intended to further refine the biological plausibility of these candidate targets by examining whether proteins prioritized through systems-level network analysis may also exhibit theoretically favorable physicochemical interactions with PTR. Given the overlapping yet mechanistically heterogeneous nature of neurodegenerative diseases [44,45], such structure-based analysis may provide an additional layer of evidence supporting the potential relevance of conserved molecular regulators. However, because docking simulations rely on static protein conformations and computational scoring functions, the obtained results should be interpreted cautiously as predictive indicators of possible molecular interactions rather than definitive confirmation of biological activity, target engagement, or therapeutic efficacy.

Docking studies demonstrated that PTR binds to SRC, ESR1, and HSP90AA1 with predicted binding energies of −4.8, −7.2, and −6.9 kcal/mol, respectively. Although molecular docking was primarily employed to generate the initial protein–ligand binding poses for subsequent molecular dynamics simulations, the corresponding interaction patterns were additionally analyzed and are described in the Supplementary Material, directly below Figure S5.

### 2.6. Molecular Dynamics Simulation

To further evaluate whether the docked PTR–protein complexes retained structural stability beyond static binding predictions, molecular dynamics (MD) simulations were performed for the highest-ranked docking conformations of SRC, ESR1, and HSP90AA1 (Figure S5). While molecular docking provides an initial theoretical estimate of binding orientation and potential affinity, it does not account for the dynamic behavior of ligand–protein interactions under time-dependent and physiologically relevant conditions. Therefore, MD simulation was employed as a complementary approach to assess the temporal stability, conformational behavior, and persistence of PTR binding within the selected target structures. By incorporating atomic-level motion and environmental flexibility, this analysis aimed to provide a more refined theoretical evaluation of whether the predicted docked complexes may remain structurally stable over time. However, as with other computational approaches, MD-derived observations should be interpreted cautiously as predictive models of dynamic interaction behavior rather than definitive confirmation of in vivo biological stability or therapeutic relevance.

**Figure 3:**
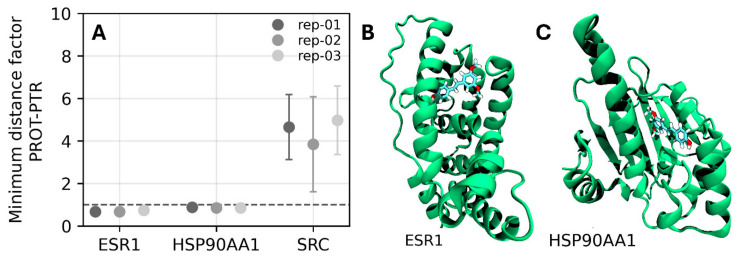
(**A**) Minimum distance factor analysis between the ESR1, HSP90AA1, and SRC binding pocket residues and PTR for the initial docking poses across 3 replicas (rep-01, rep-02, and rep-03), shown in dark gray, medium gray, and light gray, respectively. (**B**) Qualitative representation of ESR1-PTR bound complex. (**C**) Qualitative representation of HSP90AA1-PTR bound complex.

The persistence of ligand–protein interactions was initially assessed through minimum distance factor analysis, a quantitative metric that evaluates the ability of a ligand to remain in close contact with the protein binding site throughout the MD simulations (Figure 3). This parameter was calculated from the minimum ligand–protein binding site residues (Figure S5) distances sampled during the trajectories and normalized using a factor of 0.21. Results are reported as mean values with their corresponding standard deviations. Minimum distance factor values close to or lower than 1 indicate stable and persistent interactions with binding site residues, whereas values exceeding 1 suggest weaker or lose binding interactions.

The results show that all three replicas of ESR1-PTR and HSP90AA1 complexes scored minimum distance factor < 1 with standard deviations consistently below ≤0.1 (Table S16). Conversely, the SRC-PTR complex failed to meet this stability criterion, exhibiting a minimum distance factor > 1, which indicates ligand dissociation and an inability to form a stable binding interaction under dynamic conditions. Based on these refined minimum distance factor criteria applied to the investigated trajectories, ESR1-PTR and HSP90AA1-PTR complexes were retained for further detailed analyses, whereas the SRC-PTR complex was excluded.

**Figure 4:**
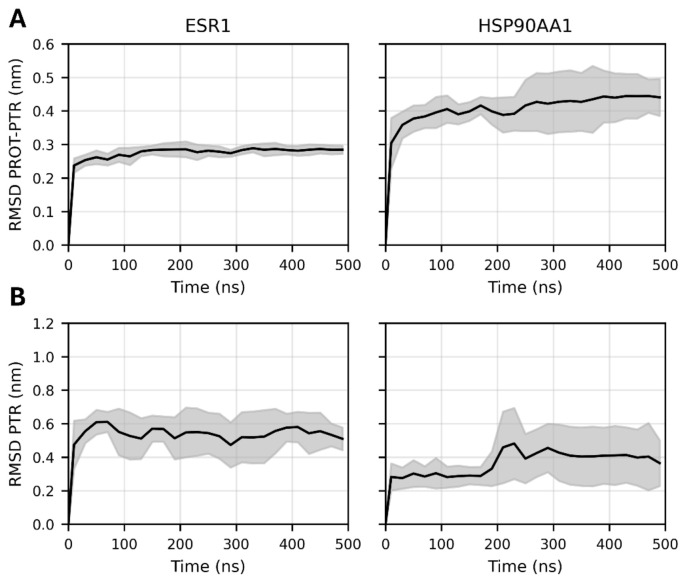
(**A**) Root mean square deviation analysis (RMSD) of ESR1-PTR and HSP90AA1-PTR protein–ligand complexes. (**B**) RMSD of the PTR ligand in both the ESR1 and HSP90AA1 complexes. Both RMSD analyses are presented as the average (black line) with the corresponding standard deviation (gray shaded area), calculated using 20 ns time windows.

To assess the conformational stability of these selected complexes, root mean square deviation (RMSD) analysis was performed on both the proteins and the ligand over the simulation trajectory. Figure 4A,B show the RMSD over the 500 ns trajectory of the protein–ligand and of the ligand complex for the selected ESR1-PTR and HSP90AA1-PTR systems. These analyses are calculated using 20 ns time windows and reported as the average with the corresponding standard deviation across the three replicas. In both cases, the RMSD reaches a plateau in the simulation and remains stable throughout the entire trajectory, indicating the absence of large conformational rearrangements and confirming the overall stability of the complex. Further proof for the conformational convergence is shown in Figure S6 in Supplementary Information File.

To characterize the binding behavior and interaction stability of the selected complexes, hydrogen bond number, buried surface area, and MM/GBSA binding free energy analyses were performed for the ESR1-PTR and HSP90AA1-PTR systems. These complementary analyses provide insight into the energetic and structural determinants governing ligand recognition and stabilization within the binding pocket throughout the simulations.

In particular, the ESR1-PTR complex showed a more favorable MM/GBSA binding free energy of −31.70 ± 2.84 kcal/mol, compared to −21.75 ± 1.51 kcal/mol for the HSP90AA1-PTR complex, suggesting a stronger energetic affinity of PTR toward ESR1. The replica-specific MM/GBSA values and standard deviations are reported in Table S17 in Supplementary Information File. Building upon this data we performed hydrogen bond analysis, where the ESR1-PTR complex maintained a slightly higher average hydrogen bond number (0.82 ± 0.26) com-pared to the HSP90AA1-PTR system (0.79 ± 0.37). In addition, buried surface area analysis revealed a slightly larger average buried surface for ESR1-PTR (3.74 ± 0.07 nm^2^) compared to HSP90AA1-PTR (3.64 ± 0.02 nm^2^), consistent with a ligand more extensive contact interface with the surrounding binding site residues.

**Figure 5:**
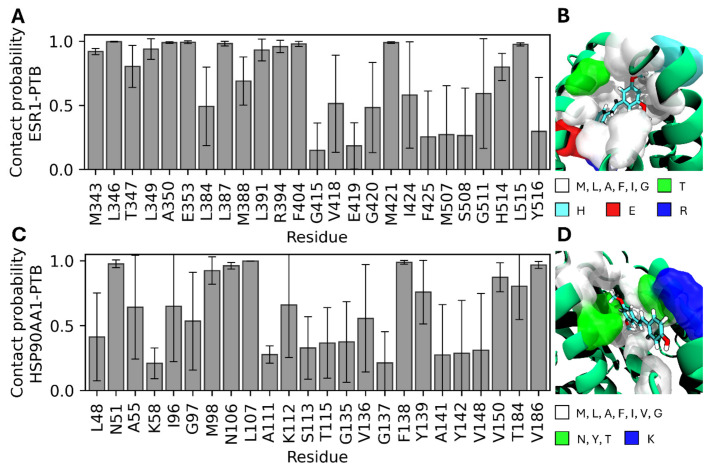
(**A**) Contact probability analysis of ESR1 residues with PTR ligand. (**B**) Qualitative representation of ESR1 residues interacting with PTR through interaction surface of the apolar (white), polar (green), polar/charged (cyan), basic (blue), and acid (red) amino acids. (**C**) Contact probability analysis of HSP90AA1 residues with PTR ligand. (**D**) Qualitative representation of HSP90AA1 residues interacting with PTR through interaction surface of the apolar (white), polar (green), and basic (blue) amino acids. For the contact probability analysis, a cut-off distance of 0.35 nm was used (**A**,**C**) and only residues with a contact probability greater than 0.50 are shown (**B**,**D**).

To gain deeper insight into the molecular determinants governing the interaction between ESR1 and HSP90AA1 proteins with PTR ligand, a contact probability analysis was performed on the selected model. The analysis was carried out using a distance cut-off of 0.35 nm, and the results are presented as a bar plot reporting the average contact probability and the associated standard deviation over the three independent replicas (Figure 5).

The contact probability analysis revealed distinct interaction patterns for the ESR1-PTR and HSP90AA1-PTR complexes. In the ESR1-PTR system, several residues displayed persistent contacts with probabilities exceeding 0.5, including M343, L346, T347, L349, A350, E353, L387, M388, L391, R394, F404, V418, M421, I424, G511, H514, and L515 (Figure 5A). A qualitative representation of the ESR1-PTR interacting conformation is shown in Figure 5B. The interaction network was predominantly composed of hydrophobic and non-polar residues, particularly leucine, methionine, isoleucine, alanine, valine, and phenylalanine residues, forming an extended hydrophobic environment surrounding the ligand. In addition, a smaller number of polar and charged residues, such as T347, E353, R394, and H524, contributed to stabilizing the complex through transient polar interactions and hydrogen bonding.

The stronger interaction profile observed for the ESR1-PTR complex is consistent with previous structural and computational studies describing the highly hydrophobic and conformationally adaptable nature of the ESR1 ligand-binding pocket [46,47]. Previous investigations demonstrated that hydrophobic interactions represent one of the major determinants governing ligand-binding affinities in ESR1, while specific polar interactions involving residues such as E353, T347, and H524 contribute to additional stabilization of the complex [46]. In agreement with these observations, the present simulations revealed that PTR establishes extensive hydrophobic contacts within the ESR1 binding cavity together with persistent polar interactions, supporting the enhanced stability and more favorable binding free energy observed for this complex.

Similarly, the HSP90AA1-PTR complex exhibited stable contacts (contact probability > 0.5) involving residues N51, A55, I96, G97, M98, N106, L107, K112, V136, F138, Y139, V150, T184, and V186 (Figure 5C). A qualitative representation of the HSP90AA1-PTR interacting conformation is shown in Figure 5D. Although hydrophobic residues were also present within the HSP90 binding pocket, the interaction interface appeared comparatively less hydrophobic and more spatially restricted than that observed in the ESR1 complex. Furthermore, the HSP90 interaction network involved a larger contribution from polar residues, including N51, N106, K112, Y139, and T184, resulting in a more heterogeneous interaction environment.

This behavior may explain the comparatively weaker binding free energy estimated for the HSP90AA1–PTR complex. Unlike ESR1, whose ligand-binding cavity is intrinsically adapted for hydrophobic ligand recognition, HSP90AA1 primarily functions as a molecular chaperone and does not possess a canonical hydrophobic steroid-like binding pocket [48,49,50]. Previous studies have shown that HSP90 interactions are often mediated through dynamic and transient recognition surfaces rather than deeply buried hydrophobic cavities [51]. Such structural differences may contribute to the reduced hydrophobic complementarity and lower interaction persistence observed for PTR within the HSP90 binding region.

The stronger interaction profile observed for ESR1-PTR may be associated with the highly hydrophobic nature of PTR, which appears to favor interaction within the predominantly apolar ESR1 binding pocket. The extensive hydrophobic contacts established with residues such as leucine, methionine, valine, isoleucine, and phenylalanine likely promote tighter packing of the ligand and contribute to the increased buried surface area observed during the simulations. This enhanced hydrophobic complementarity may also explain the more favorable MM/GBSA binding free energy obtained for the ESR1 complex.

### 2.7. Role of ESR1 and HSP90AA1 in Neurodegenerative Diseases

ESR1 encodes estrogen receptor alpha (ERα), a receptor involved in estrogen-mediated signaling in the brain. This pathway has been repeatedly associated with neuronal survival and cognitive function. In AD, ESR1 dysfunction has been described as a potential upstream event contributing to neuroinflammation and pyroptosis, which may aggravate disease progression [52]. Other data suggest that ESR1 may participate in broader regulatory networks involved in neurogenesis, neuronal survival, β-amyloid metabolism and memory-related mechanisms, including pathways associated with BDNF and NTF3 [53]. Li et al. [54] provided genetic evidence supporting the involvement of ESR1 in cognitive impairment. In their population-based study, the low-frequency ESR1 variant rs9340803 was associated with both late-onset AD and mild cognitive impairment. This variant was also linked to reduced ESR1 expression, altered cholesterol-related parameters, and Aβ-associated changes. Tecalco-Cruz et al. discussed ESR1-dependent estrogen signaling as an important mechanism involved in maintaining cognitive health and suggested that reduced estrogen levels during aging, particularly in women, may increase susceptibility to AD-related neurodegeneration [55]. Pietrzak-Wawrzyńska et al. [56] demonstrated that in experimental models of Alzheimer’s disease, selective activation of non-nuclear estrogen receptor signaling increased membrane ESR1/ERα and ESR2/ERβ levels, enhanced autophagy-related cellular responses, and reduced Aβ-induced neuronal damage. The relevance of ESR1 is not limited to AD. Chowdhury et al. [57] reported in a network medicine study focused on PD and ALS, ESR1, EGFR, and SRC were identified as key hub–bottleneck genes, while estrogen signaling was significantly enriched. Interestingly, ESR1 showed opposite expression patterns in these diseases, being upregulated in ALS and downregulated in PD, which suggests that estrogen-related signaling may be regulated in a disease-specific manner [57].

HSP90AA1 encodes the inducible HSP90α chaperone, which is involved in protein folding, stress responses and maintenance of proteostasis. This is highly relevant to neurodegenerative diseases, where the accumulation of misfolded or aggregated proteins is a central pathological feature. In AD hippocampal tissue, members of the Hsp90 chaperone family were associated with neurodegeneration and astrogliosis, and changes in HSP90-related proteins were linked with Aβ and tau homeostasis through chaperone-mediated autophagy [58]. Astillero-Lopez et al. [59] identified HSP90AA1, together with PTK2B and ANXA2, as proteins potentially involved in the maintenance of synaptic homeostasis in the human entorhinal cortex in AD. Their analysis linked these proteins with synaptic function, Aβ/tau pathology, and neuron–glia interactions, suggesting that HSP90AA1 may contribute to AD-related synaptic alterations through mechanisms involving microglial cells. Another study identified HSP90AA1 as a potential AD biomarker related to manganese metabolism, with reduced expression confirmed in AD samples [60]. At the mechanistic level, HSP90AA1 may be particularly important for autophagy-dependent quality control. HSP90AA1 was shown to regulate TFEB nuclear localization and autophagic activity, indicating a possible role in the clearance of damaged organelles and misfolded proteins [61]. This observation is relevant because impaired autophagy is considered an important component of AD pathology and is also implicated in other chronic neurodegenerative diseases, including PD, HD and ALS [56,62]. In PD models HSP90AA1 was further connected with oxidative stress regulation. BDNF reduced rotenone-induced oxidative stress through the HSP90AA1/NRF2 pathway, suggesting that HSP90AA1 may contribute to antioxidant defense mechanisms in dopaminergic neurodegeneration [63].

### 2.8. Summary and Perspectives

Our previous experimental studies [20,21] demonstrated that PTR, particularly in amorphous solid dispersions, exhibits improved solubility, enhanced antioxidant activity, neuroprotective effects, cholinesterase-inhibitory activity, and increased permeability across biological membranes. These findings provide an experimental basis for the biological relevance of PTR and support further investigation of its potential neuroprotective mechanisms.

The present in silico analysis extends these observations by prioritizing candidate molecular targets and biological processes that may be associated with PTR activity in neurodegenerative disease contexts. In particular, the enrichment of GO Biological Process terms related to oxidative stress responses, programmed cell death/apoptotic processes, immune-system regulation/neuroinflammation, MAPK/ERK- and PI3K/AKT-associated signaling, ion and calcium transport, and lipid-, steroid-, or hormone-associated regulation is consistent with the previously demonstrated antioxidant and neuroprotective properties of PTR. Moreover, the predicted interactions of PTR with ESR1 and HSP90AA1, supported by molecular docking and molecular dynamics simulations, provide structural hypotheses that may help explain the potential multitarget activity of PTR. However, it should be emphasized that the findings of the present study are based exclusively on computational approaches and theoretical models. Therefore, the identified targets, enriched biological processes, and predicted protein–ligand interactions should be interpreted as hypothesis-generating rather than definitive mechanistic evidence.

Future studies should focus on experimental validation of the prioritized targets and biological processes using appropriate biochemical assays, cellular models, and animal models of neurodegenerative diseases. In particular, validation of PTR effects on ESR1- and HSP90AA1-associated signaling, together with selected neurodegenerative and neuroinflammatory markers, would be an important next step. Further computational refinement, including more advanced free energy calculations, may also improve the understanding of PTR–target interactions. Overall, the present study provides a systems-level theoretical framework for future experimental investigations of PTR as a potential multitarget neuroprotective agent.

## 3. Materials and Methods

### 3.1. Collection of Potential Targets of Pterostilbene

Potential molecular targets of PTR were identified using a combination of web-based target prediction tools, including Swiss Target Prediction (http://www.swisstargetprediction.ch/, accessed on 18 November 2025) [64], SuperPred (https://prediction.charite.de/, accessed on 18 November 2025) [65], TargetNet (http://targetnet.scbdd.com/, accessed on 18 November 2025) [66], and PharmMapper (http://lilab-ecust.cn/pharmmapper/, accessed on 18 November 2025) [67]. Canonical SMILES of PTR was uploaded to Swiss Target Prediction (Homo sapiens, probability ≥ 0.1), SuperPred (known strong binders and additionally predicted targets of probability ≥ 80%) and TargetNet (ECFP4 fingerprints, AUC ≥ 0.7, probability ≥ 0.6). Molecular structure of PTR in sdf format was retrieved from PubChem (PubChem CID: 5281727; website: https://pubchem.ncbi.nlm.nih.gov/, accessed on accessed 22 November 2025). Sdf file was uploaded to PharmMapper with limitation to “Human Protein Targets”and z’-score ≥ 0.9. All predicted targets were standardized to UniProtKB identifiers Uniprot ID, https://www.uniprot.org/help/uniprotkb, accessed on 18 November 2025) [68], and de-duplicated, and non-human proteins were excluded, retaining only Homo sapiens targets.

### 3.2. Disease Target Collection

The GeneCards database [31,32] (Relevance Score ≥ 5.0; https://www.genecards.org/, accessed on 23 November 2025) was used to identify disease-associated targets for major neurodegenerative disorders. Target retrieval was conducted using the keywords “Alzheimer’s disease”, “Parkinson’s disease”, “Huntington’s disease”, and “amyotrophic lateral sclerosis”. Although GeneCards integrates evidence from numerous curated biomedical databases, the resulting disease-associated gene sets may still be biased toward highly studied genes and should therefore be interpreted as putative disease-related targets requiring further experimental validation.

### 3.3. Selection of Common Targets

Intersection analysis between disease-associated targets and predicted targets for PTR was conducted for each neurodegenerative disease using the Draw Venn Diagram tool (http://bioinformatics.psb.ugent.be/webtools/Venn/, accessed on 22 December 2025). A summary of the input dataset sizes used for these analyses is presented in Table 1, while the resulting shared target sets were subsequently identified for downstream network pharmacology analyses.

### 3.4. Construction of Protein–Protein Interaction (PPI) Network

Shared targets identified through intersection analysis between disease-associated targets and predicted pterostilbene (PTR) targets for each neurodegenerative disease (AD, PD, HD, and ALS) were used as input datasets for PPI network construction. The corresponding shared target files (“PTR-AD_venn_result.txt”, “PTR-PD_venn_result.txt”, “PTR-HD_venn_result.txt”, and “PTR-ALS_venn_result.txt”) were imported into the Search Tool for the Retrieval of Interacting Genes (STRING) database (version 12.0; https://string-db.org, accessed on 5 January 2026) [69] for protein–protein interaction analysis in Homo sapiens. The full STRING network option was applied to capture both functional and physical protein associations. Networks were generated using the highest confidence interaction score threshold (≥0.9) [40,70], with active interaction sources restricted to text mining, experimental evidence, and curated databases. Edge meaning was based on evidence, and disconnected nodes were excluded from network visualization.

The resulting giant PPI networks were exported to Cytoscape (version 3.10.3) [71] for visualization and topological analysis. For each disease-specific network, Cytoscape session files (“_PPI_giant_network.cys”) and giant network visualizations (“[giant network] .png”) were generated. Network topology was evaluated using the CytoNCA plugin [72], where networks were analyzed as undirected and unweighted graphs according to default settings. Prior to centrality analysis, parallel edges and self-loops were removed to reduce redundancy. Degree centrality (DC), betweenness centrality (BC), closeness centrality (CC), and eigenvector centrality (EC) were calculated for all nodes, and full ranked outputs were exported as “_Results ranked by Degree.txt” and “_Results ranked by Betweenness.txt”, containing raw DC, BC, CC, and EC values for all proteins.

Hub subnetworks were defined as the top 5% of nodes ranked by DC, while bottleneck subnetworks were defined as the top 5% ranked by BC. This approach is widely applied in network biology as a practical method for prioritizing candidate proteins with potential structural or regulatory importance within complex interaction systems [41,42,43,44]. Corresponding subnetwork visualizations were generated as “[hub network]_top5%_Results ranked by Degree.png” and “[bottleneck network]_top5%_Results ranked by Betweenness.png”. Overlap between hub and bottleneck nodes was subsequently identified and summarized in “*_top5%_Results_summary.xlsx” files (PTR-AD, PTR-PD, PTR-HD, PTR-ALS), where overlapping proteins were highlighted to facilitate prioritization of candidate key targets.

All raw data files, processed network outputs, Supplementary Result Files, and associated documentation are publicly available in the open-access RepOD repository (https://doi.org/10.18150/HNUSRO). Proteins consistently identified within overlapping hub–bottleneck subnetworks were considered representative candidate targets for subsequent molecular docking and molecular dynamics simulations.

### 3.5. Functional Enrichment Analysis

GO Biological Process (GO BP) enrichment analysis was performed for the disease-specific shared PTR–disease target sets corresponding to the giant PPI networks generated for PTR-AD, PTR-HD, PTR-PD, and PTR-ALS. Enrichment analysis was conducted using the STRING database (version 12.0; http://string-db.org, accessed on 8 January 2026) [69] for Homo sapiens. The PPI networks were constructed using a high-confidence interaction score threshold (≥0.9), and GO BP enrichment was performed directly within the STRING platform using the default whole-genome background. Significantly enriched GO BP terms were identified based on STRING-integrated statistical analysis with Benjamini–Hochberg false discovery rate (FDR) correction. Terms with FDR < 0.05 were considered statistically significant.

To improve the specificity and interpretability of the enrichment results, the STRING-derived GO BP outputs were subjected to a multistep filtering and redundancy-reduction procedure. First, GO BP terms associated with fewer than 3 or more than 50 genes were excluded, according to previously described approaches aimed at reducing the contribution of overly narrow as well as highly general and non-specific functional categories [73,74,75]. Thus, only GO BP terms fulfilling the criterion 3 ≤ B ≤ 50 were retained, where B denotes the number of input-network genes associated with a given GO BP term. Next, the remaining enriched GO BP terms were ranked according to FDR-adjusted significance, and the top 20% most significantly enriched terms were retained as an intermediate pool for further evaluation.

From this intermediate pool, GO BP terms directly related to neurodegeneration- and neuroprotection-relevant mechanisms were selected for further analysis. This focused set of GO BP terms was subsequently subjected to semantic similarity-based redundancy reduction using REVIGO (version 1.8.2) [33].

For REVIGO analysis, GO identifiers together with their corresponding STRING-derived FDR-adjusted values were used as input. REVIGO analysis was performed using the Homo sapiens Gene Ontology database, the SimRel semantic similarity measure, and the medium similarity setting. REVIGO outputs were used to identify representative non-redundant GO BP terms and semantically related term clusters. Representative terms were selected based on REVIGO dispensability scores, with lower dispensability values indicating less redundant and more representative biological processes.

The full STRING GO BP enrichment outputs, filtered GO BP datasets, REVIGO-reduced tables, and top-ranked representative GO BP summaries were retained for each disease-specific dataset. To ensure traceability of the multistep filtering procedure, the input data used at each filtering stage were compiled in dedicated .xlsx files. All raw and processed files are available in the open-access RepOD repository within the “Enrichment analysis.zip” archive (https://doi.org/10.18150/HNUSRO), ensuring transparency and reproducibility of the enrichment analyses.

### 3.6. Molecular Docking

UCSF Chimera (version 1.18) [76] was employed for the preparation of target proteins prior to molecular docking. The three-dimensional structures of the target proteins: C-SRC (PDB ID: 1A07), ESR1 (PDB ID: 1ERR), and HSP90AA1 (PDB ID: 1OSF) were obtained from the RCSB Protein Data Bank (PDB; https://www.rcsb.org/, accessed on 11 January 2026) as original .pdb files. For each target, the corresponding repository (https://doi.org/10.18150/HNUSRO) folders (“1A07_SRC”, “1ERR_ESR1”, and “1OSF_HSP90AA1”) within “molecular docking.zip” contain the complete docking workflow, including original protein structures, intermediate preparation files, docking configuration files, and final docking outputs. All co-crystallized ligands and water molecules were removed from the protein structures (“_naked.pdb”), and only conformations with the highest occupancy were retained. Missing side chains in protein structures were reconstructed using the Dunbrack 2010 rotamer library [77]. The reconstructed residues for C-SRC were Lys120, Gln136, Glu196, and Asp219 in chain A; Asn212 in chain B; and Ser22 in chain C. For ESR1, the reconstructed residues in chain A were Tyr331, Asp332, Glu397, Lys416, Glu419, Glu470, Glu471, Lys472, Arg477, Lys492, and Glu542, whereas no incomplete side chains were identified in HSP90AA1. Detailed information about them is provided in the corresponding target-specific “log.txt” files within the “molecular docking.zip” repository archive. Next, hydrogen atoms were added to satisfy valence requirements, and Gasteiger partial charges were assigned to the protein models.

The PTR ligand structure was retrieved from PubChem (CID: 5281727) in .sdf format (“pterostilbene_CID_5281727.sdf”) and prepared in UCSF Chimera (version 1.18) [76]. Ligand geometry optimization was performed using the Minimize Structure module to relieve steric clashes and generate an energetically favorable conformation prior to docking, resulting in the minimized ligand file (“PTR_minimized.mol2”). Following ligand preparation and docking setup, the processed ligand was exported in both PDB and PDBQT formats as “docking.ligand.pdb” and “docking.ligand.pdbqt”, respectively, to ensure compatibility with AutoDock Vina 4.0 and facilitate downstream structural analyses.

Molecular docking simulations were performed using AutoDock Vina implemented in UCSF Chimera (version 1.18). Prior to docking, polar hydrogens were added, and Gasteiger charges were assigned to both the receptor and ligand. Non-polar hydrogens and lone pairs were merged, while water molecules were removed. Grid box parameters were defined in Chimera to encompass the native ligand-binding regions of the original crystal structures, based on the spatial position of the co-crystallized ligands. Docking box dimensions and coordinates were documented in target-specific configuration files (“docking.conf”) and visualized using binding-site reference images (e.g., “SRC_box.png”). For C-SRC, the grid center was set to (44, 17, 22) with dimensions of 22 × 24 × 18 Å. For ESR1, the grid center was set to (69, 35, 80) with dimensions of 19 × 40 × 36 Å. For HSP90AA1, the grid center was set to (76, −30, 65) with dimensions of 21 × 27 × 35 Å. Docking calculations were performed using default Vina parameters, with the number of binding modes set to 9, exhaustiveness to 8, and maximum energy difference to 3 kcal/mol. For each target protein, ten docking poses were generated, and the complex with the lowest predicted binding energy was selected as the representative conformation for subsequent interaction analysis, structural visualization, and downstream molecular dynamics simulations (e.g., “*_pose1.pdb”). 3D and 2D visualizations of the ligand–protein interactions were generated using BIOVIA Discovery Studio Visualizer (version 25.1.0.24284).

### 3.7. Molecular Dynamics Simulation

To evaluate the stability and dynamic behavior of the selected protein–ligand complexes under physiologically relevant conditions, all systems were subjected to all-atom molecular dynamics (MD) simulations. The 3D structure of pterostilbene (PTR) was obtained from PubChem repository [78] and partial atomic charges were derived using the abcg2 charge method [79] with a total charge of 0. Ligand topologies were generated using the ACPYPE [80] tool in combination with the General Amber Force Field 2 (GAFF2) [81], following protocols previously applied in the literature [82,83].

Each protein–ligand complex was placed at the center of a dodecahedral simulation box with a minimum distance of 1.5 nm between periodic images and solvated using explicit water molecules. Sodium and chloride ions were introduced to neutralize the systems and mimic physiological ionic strength (150 mM), resulting in simulation systems containing approximately 25,000, 40,000, and 48,000 interacting particles for the SRC-PTR, ESR1-PTR, and HSP90-PTR complexes, respectively. All systems were energy-minimized using the steepest descent algorithm for 2000 steps. Subsequently, equilibration was carried out under position restraints by gradually heating the systems to 310 K (τ_t_ = 1 ps) over 200 ps in the NVT ensemble, followed by 200 ps of equilibration in the NPT ensemble at 1 atm (τ_p_ = 5 ps). Temperature and pressure were controlled using the V-rescale [84] and C-rescale [85] coupling schemes, respectively, during both equilibration and production phases.

Production MD simulations were subsequently performed in the NPT ensemble at a constant temperature of 310 K (τ_t_ = 1 ps) and pressure of 1 atm (τ_p_ = 5 ps). A 4 fs integration time step was achieved through the implementation of a hydrogen mass repartitioning (HMR) scheme [86], in which the minimum atomic mass was scaled by a factor of 3, in combination with LINCS constraints [87]. The Amber ff19SB [88] force field was used to describe the protein, while the TIP3P [89] model was employed for water molecules. All simulations were performed using GROMACS 2026.1 [90,91]. Trajectory visualization and inspection were carried out using Visual Molecular Dynamics (VMD) [92]. Each protein–ligand complex was simulated for 500 ns in three independent replicas.

The binding free energy estimations were performed using the Molecular Mechanics Generalized Born Surface Area (MM/GBSA) approach [93] with gmx_MMPBSA tool [94]. Calculations were carried out on all replicas using 50 evenly spaced frames extracted from the last 50 ns of each trajectory. The generalized Born model was applied with GB-Neck2 (igb = 8) [95] was applied to estimate the polar solvation energy. An implicit solvent salt concentration of 0.15 M was applied to mimic physiological ionic conditions, while the temperature was maintained at 310 K, consistent with the conditions employed during the production MD simulations.

### 3.8. Limitations

Several limitations of the present study should be acknowledged. First, disease-associated gene sets were obtained from the GeneCards database using a relevance score threshold of ≥5. Although this threshold has been previously applied in network pharmacology studies, no universally accepted GeneCards relevance-score cut-off currently exists. Different studies have employed thresholds ranging from 5 to 30 (e.g., >5 [96,97,98,99], >10 [100,101,102], >20 [103], >30 [104,105], as well as alternative ranking-based approaches such as median-score filtering [106,107] or selection of top-ranked genes [108]. Consequently, the composition of disease-associated gene sets may vary depending on the selected filtering criteria. Moreover, GeneCards integrates information from heterogeneous biomedical sources, and gene-disease associations may reflect different levels of evidence. Therefore, GeneCards-derived datasets may be affected by annotation density and publication bias toward extensively studied genes. This limitation was further illustrated by the additional threshold-sensitivity check performed in the present study. Increasing the GeneCards relevance-score threshold from ≥5 to ≥10 and ≥20 reduced the number of overlapping PTR–disease targets across all four analyzed neurodegenerative disorders (Table S18 in the Supplementary Materials). These findings indicate that the generated PPI networks, hub and bottleneck identification, and enrichment analysis results are threshold-dependent. Therefore, the identified key targets should be interpreted as prioritized candidate proteins rather than definitive disease-driving factors.

Second, the target prediction workflow relied on computational tools and publicly available databases, each characterized by its own prediction algorithms, confidence metrics, and underlying datasets. Although a multi-platform strategy was employed to improve robustness, false-positive and false-negative predictions cannot be completely excluded. In addition, full cross-validation of all disease-associated targets against independent disease databases such as OMIM, DisGeNET, CTD, or DrugBank was not performed at this stage. Therefore, the resulting PTR–disease target overlap should be considered a hypothesis-generating dataset requiring further validation.

Third, PPI network construction and centrality-based prioritization are inherently dependent on the completeness and quality of the input datasets and interaction databases. Highly studied proteins may be overrepresented in interaction networks, which can influence network topology, centrality values, and the identification of hub or bottleneck nodes. Thus, network-derived prioritization should be interpreted as a computational ranking strategy rather than direct evidence of biological causality.

Fourth, molecular docking and molecular dynamics simulations provide structural hypotheses regarding potential PTR–protein interactions but do not experimentally confirm target engagement or binding affinity. Docking scores are approximate and may be influenced by protein structure selection, ligand conformation, protonation states, and scoring-function limitations. Similarly, although molecular dynamics simulations improve the structural interpretation of protein–ligand stability, they remain model-dependent and cannot replace biophysical or cellular validation.

Finally, all findings presented in this study are based on in silico methodologies, including target prediction, network pharmacology, enrichment analysis, molecular docking, and molecular dynamics simulations. While these approaches provide valuable mechanistic hypotheses and enable systematic prioritization of candidate targets, experimental validation is required to confirm the biological relevance of the identified targets, pathways, and predicted protein–ligand interactions. Future studies should therefore verify the effects of PTR on prioritized targets, particularly ESR1 and HSP90AA1, together with selected neurodegenerative and neuroinflammatory markers in appropriate in vitro and/or in vivo models.

## 4. Conclusions

In the present study, an integrative network pharmacology, molecular docking, and molecular dynamics (MD) simulation framework was applied to explore the potential multitarget mechanisms through which pterostilbene (PTR) may be relevant to neurodegenerative diseases, including Alzheimer’s disease, Parkinson’s disease, Huntington’s disease, and amyotrophic lateral sclerosis. Systems-level network analyses suggested that PTR-associated targets may be distributed across shared molecular architectures relevant to multiple neurodegenerative phenotypes rather than exclusively disease-specific pathways.

Redundancy-reduced GO Biological Process enrichment analysis indicated that these shared targets may be associated with biological processes relevant to neurodegeneration and neuroprotection, including immune-system regulation and neuroinflammation, oxidative stress and reactive oxygen species-related responses, programmed cell death and apoptotic processes, MAPK/ERK- and PI3K/AKT-related signaling, ion and calcium transport, and lipid-, steroid-, or hormone-associated regulation. PPI network topology suggested C-SRC, ESR1, and HSP90AA1 as recurrently prioritized hub–bottleneck proteins; however, further structural interpretation was focused primarily on ESR1 and HSP90AA1, which were selected for detailed molecular dynamics-based evaluation.

MD analyses suggested favorable binding of PTR to key protein targets identified through network pharmacology, including HSP90AA1 and ESR1. In particular, minimum distance factor analyses demonstrated persistent ligand interaction within the binding pockets across all simulation replicas, while RMSD analyses confirmed the structural stability of both the proteins and the bound ligand throughout the 500 ns trajectories.

Further characterization through hydrogen bond number, buried surface area, MM/GBSA binding free energy calculations, and contact probability analyses revealed stable and persistent interactions between PTR and both target proteins. Notably, the ESR1-PTR complex displayed a more favorable interaction profile compared to HSP90AA1-PTR, characterized by stronger binding free energy estimations, slightly higher hydrogen bond persistence, and increased buried surface area. Contact probability analyses further showed that PTR established an extended interaction network with predominantly hydrophobic residues within the ESR1 binding pocket, promoting enhanced interaction stability.

These findings suggest that ESR1 represents the most favorable predicted target for PTR among the analyzed systems, supporting the potential involvement of estrogen receptor-mediated signaling pathways in the neuroprotective activity of PTR. HSP90AA1 also remained a structurally supported candidate target, suggesting that PTR may additionally interact with protein homeostasis- and stress-response-related mechanisms relevant to neurodegenerative processes. Although these results do not constitute direct experimental validation of binding, they provide computationally derived structural hypotheses regarding the potential molecular basis underlying PTR activity.

In summary, the network pharmacology, molecular docking, and MD simulation results collectively suggest that PTR may potentially influence neurodegeneration-relevant molecular systems through coordinated interactions with multiple targets and signaling pathways associated with metabolic regulation, stress responses, cellular homeostasis, and conserved intracellular signaling. However, these findings are derived exclusively from in silico analyses and predictive computational models, and therefore should be interpreted cautiously as a hypothesis-generating framework rather than definitive evidence of direct neuroprotective mechanisms. Accordingly, the present study may provide a systems-level theoretical foundation for future in vitro and in vivo investigations aimed at validating the biological relevance, mechanistic significance, and potential translational implications of PTR in neurodegenerative disease contexts, particularly with respect to ESR1- and HSP90AA1-associated signaling and selected neurodegenerative or neuroinflammatory markers.

## Figures and Tables

**Table 1:** Summary of input dataset sizes used for intersection analyses between PTR and neurodegenerative disease-associated targets. Detailed target lists for PTR, AD, HD, PD, and ALS are provided in the Supplementary Archive “Venn_results.zip” (File: “Venn diagram_input.xlsx”; tabs: PTR, AD, HD, PD, and ALS), available in the online repository (https://doi.org/10.18150/HNUSRO).

PTR–Disease	Name	Number of Elements	Number of Unique Elements	Overall Number of Unique Elements
PTR-AD	AD	6381	6375	6409
	PTR	215	215	
PTR-HD	HD	3731	3727	3814
	PTR	215	215	
PTR-PD	PD	5319	5319	5534
	PTR	215	215	
PTR-ALS	ALS	3491	3489	3595
	PTR	215	215	

## Data Availability

Files are deposited in an open database—RepOD: https://doi.org/10.18150/HNUSRO.

## Supplementary Materials

The following supporting information can be downloaded at: https://www.mdpi.com/article/10.3390/ph19071053/s1, Figure S1: PPI network analysis of PTR-AD: a giant network obtained after removing disconnected nodes. The figure is available as a high-resolution bitmap (.png format; “[giant network] PTR-AD in the online repository (https://doi.org/10.18150/HNUSRO) in PPI network_results.zip. Figure S2: PPI network analysis of PTR-HD: a giant network obtained after removing disconnected nodes. The figure is available as a high-resolution bitmap (.png format; “[giant network] PTR-HD”) in the online repository (https://doi.org/10.18150/HNUSRO) in PPI network_results.zip. Figure S3: PPI network analysis of PTR-PD: a giant network obtained after removing disconnected nodes. The figure is available as a high-resolution bitmap (.png format; “[giant network] PTR-PD”) in the online repository (https://doi.org/10.18150/HNUSRO) in PPI network_results.zip. Figure S4. PPI network analysis of PTR-ALS: a giant network obtained after removing disconnected nodes. The figure is available as a high-resolution bitmap (.png format; “[giant network] PTR-ALS”) in the online repository (https://doi.org/10.18150/HNUSRO) in PPI network_results.zip. Figure S5. Visualization of docking results of best ranked pose of key interactions in the binding pocket of SRC (PDB ID: 1A07, predicted binding energy of −4.8 kcal/mol), ESR1 (PDB ID: 1ERR, predicted binding energy of −7.2 kcal/mol), and HSP90AA1 (PDB ID: 1OSF, predicted binding energy of −6.9 kcal/mol) with pterostilbene (CID: 5281727). (a) 3D representation of PTR-SRC, (b) 2D representation of PTR-SRC, (c) 3D representation of PTR-ESR1, (d) 2D representation of PTR-ESR1, (e) 3D representation of PTR-HSP90AA1, (f) 2D representation of PTR-HSP90AA1. Legend: ALA—alanine, ARG—arginine, ASN—asparagine, ASP—aspartic acid, GLU—glutamic acid, GLY—glycine, HIS—histidine, ILE—isoleucine, LEU—leucine, LYS—lysine, MET—methionine, PHE—phenylalanine, SER—serine, THR—threonine, TRP—tryptophan, TYR—tyrosine, VAL—valine. Protein–ligand interactions are highlighted in green, whereas the cyan surface denotes the solvent-accessible surface area (SASA). Figure S6. Radius of gyration (RG) of ESR1-PTR and HSP90AA1-PTR protein-ligand systems. Both RG analyses are presented as the average (black line) with the corresponding standard deviation (grey shaded area), calculated using 20 ns time windows. Table S1. Potential molecular targets of pterostilbene (PTR). Target identifiers are provided according to the UniProtKB database (UniProt IDs). Yellow highlighted cells indicate duplicate targets between sets. SwissTarget, SuperPred, TargNet, PharmMapper—web-based target prediction tools. All data presented in this table are available as an .xlsx file (‘Potential molecular targets of pterostilbene’, sheet ‘marked duplicates’) in the online repository (https://doi.org/10.18150/HNUSRO). Table S2. List of 215 unique PTR targets. Target identifiers are provided according to the UniProtKB database (UniProt IDs). SwissTarget, SuperPred, TargNet, PharmMapper—web-based target prediction tools. All data presented in this table are available as an .xlsx file (‘Potential molecular targets of pterostilbene’, sheet ‘no duplicates’) in the online repository (https://doi.org/10.18150/HNUSRO). Table S3. Shared targets of PTR and neurodegenerative diseases identified by Venn diagram analysis. Target identifiers are provided according to the UniProtKB database (UniProt IDs). Legend: PTR—pterostilbene, AD—Alzheimer’s disease, HD—Huntington’s disease, PD—Parkinson’s disease, ALS—amyotrophic lateral sclerosis. All data presented in this table are available as an .xlsx file (‘Venn diagram_intersection’, sheet ‘summary’) in the online repository (https://doi.org/10.18150/HNUSRO). Table S4: Redundancy-reduced GO Biological Process enrichment results for PTR-AD obtained using REVIGO semantic clustering. Legend: In highlighted REVIGO clusters, representative terms are shown in bold, whereas semantically related terms assigned to the same representative cluster are indicated using the same background color. TermID denotes the GO identifier, Name indicates the biological process, and Value corresponds to the input statistical value used for REVIGO analysis. LogSize and Frequency describe the relative size and occurrence frequency of each GO term in the Gene Ontology database, whereas Uniqueness and Dispensability indicate semantic distinctiveness and redundancy, respectively. Lower dispensability values indicate less redundant and more representative GO terms. Table S5. Redundancy-reduced GO Biological Process enrichment results for PTR-HD obtained using REVIGO semantic clustering. Legend: In highlighted REVIGO clusters, representative terms are shown in bold, whereas semantically related terms assigned to the same representative cluster are indicated using the same background color. TermID denotes the GO identifier, Name indicates the biological process, and Value corresponds to the input statistical value used for REVIGO analysis. LogSize and Frequency describe the relative size and occurrence frequency of each GO term in the Gene Ontology database, whereas Uniqueness and Dispensability indicate semantic distinctiveness and redundancy, respectively. Lower dispensability values indicate less redundant and more representative GO terms. Table S6: Redundancy-reduced GO Biological Process enrichment results for PTR-PD obtained using REVIGO semantic clustering. Legend: In highlighted REVIGO clusters, representative terms are shown in bold, whereas semantically related terms assigned to the same representative cluster are indicated using the same background color. TermID denotes the GO identifier, Name indicates the biological process, and Value corresponds to the input statistical value used for REVIGO analysis. LogSize and Frequency describe the relative size and occurrence frequency of each GO term in the Gene Ontology database, whereas Uniqueness and Dispensability indicate semantic distinctiveness and redundancy, respectively. Lower dispensability values indicate less redundant and more representative GO terms. Table S7: Redundancy-reduced GO Biological Process enrichment results for PTR-ALS obtained using REVIGO semantic clustering. Legend: In highlighted REVIGO clusters, representative terms are shown in bold, whereas semantically related terms assigned to the same representative cluster are indicated using the same background color. TermID denotes the GO identifier, Name indicates the biological process, and Value corresponds to the input statistical value used for REVIGO analysis. LogSize and Frequency describe the relative size and occurrence frequency of each GO term in the Gene Ontology database, whereas Uniqueness and Dispensability indicate semantic distinctiveness and redundancy, respectively. Lower dispensability values indicate less redundant and more representative GO terms. Table S8: Top representative GO Biological Process terms for PTR-AD selected after REVIGO semantic clustering. Representative terms within highlighted clusters are shown in bold, while semantically related terms assigned to the same representative cluster are marked with the same background color. The No. column indicates the ranked representative term or cluster, TermID denotes the GO identifier, and Name indicates the biological process. Table S9: Top representative GO Biological Process terms for PTR-HD selected after REVIGO semantic clustering. Representative terms within highlighted clusters are shown in bold, while semantically related terms assigned to the same representative cluster are marked with the same background color. The No. column indicates the ranked representative term or cluster, TermID denotes the GO identifier, and Name indicates the biological process. Table S10: Top representative GO Biological Process terms for PTR-PD selected after REVIGO semantic clustering. Representative terms within highlighted clusters are shown in bold, while semantically related terms assigned to the same representative cluster are marked with the same background color. The No. column indicates the ranked representative term or cluster, TermID denotes the GO identifier, and Name indicates the biological process. Table S11: Top representative GO Biological Process terms for PTR-ALS selected after REVIGO semantic clustering. Representative terms within highlighted clusters are shown in bold, while semantically related terms assigned to the same representative cluster are marked with the same background color. The No. column indicates the ranked representative term or cluster, TermID denotes the GO identifier, and Name indicates the biological process. Table S12: Hub network (Top 5% of degree value) and bottleneck network (Top 5% of betweenness value—BC) of pterostilbene-Alzheimer’s disease PPI giant network. STRING protein identifiers (STRING name) were converted to canonical gene symbols (display name). Green-highlighted cells indicate proteins common to both the hub and bottleneck networks. Red-highlighted cells indicate proteins without connections, which were excluded from further analysis. All data presented in this table are available as an .xlsx file (‘PTR-AD_top5%_Results_summary’) in the online repository (https://doi.org/10.18150/HNUSRO, file: ‘PPI network_results.zip’). Table S13: Hub network (Top 5% of degree value) and bottleneck network (Top 5% of betweenness value—BC) of pterostilbene-Huntington’s disease PPI giant network. STRING protein identifiers (STRING name) were converted to canonical gene symbols (display name). Green-highlighted cells indicate proteins common to both the hub and bottleneck networks. All data presented in this are available as an .xlsx file (‘PTR-HD_top5%_Results_summary’) in the online repository (https://doi.org/10.18150/HNUSRO, file: ‘PPI network_results.zip’). Table S14: Hub network (Top 5% of degree value) and bottleneck network (Top 5% of betweenness value—BC) of pterostilbene-Parkinson’s disease PPI giant network. STRING protein identifiers (STRING name) were converted to canonical gene symbols (display name). Green-highlighted cells indicate proteins common to both the hub and bottleneck networks. All data presented in this table are available as an .xlsx file (‘PTR-PD_top5%_Results_summary’) in the online repository (https://doi.org/10.18150/HNUSRO, file: ‘PPI network_results.zip’). Table S15: Hub network (Top 5% of degree value) and bottleneck network (Top 5% of betweenness value—BC) of pterostilbene-amyotrophic lateral sclerosis PPI giant network. STRING protein identifiers (STRING name) were converted to canonical gene sym-bols (display name). Green-highlighted cells indicate proteins common to both the hub and bottleneck networks. All data presented in this table are available as an .xlsx file (‘PTR-ALS_top5%_Results_summary’) in the online repository (https://doi.org/10.18150/HNUSRO, file: ‘PPI network_results.zip’). Table S16: Average and standard deviation of the minimum distance factor analysis between the ESR1, HSP90, and SCR binding pocket residues and pterostilbene (PTR) for the initial docking poses across 3 replicas. Table S17: Average and standard deviation of the MM/GBSA between the ESR1 and HSP90 proteins and pterostilbene (PTR) across 3 replicas. Table S18: Threshold-sensitivity check showing the number of overlapping PTR–disease targets identified using different GeneCards relevance-score cutoffs (>5, >10, and >20). The underlying data are provided in the RepOD repository (https://doi.org/10.18150/HNUSRO) in the file “Threshold-sensitivity check GeneCards.tab”. Figures S1–S6 and Tables S1–S18 are in Supplementary Materials. All input files used in the theoretical analyses, as well as all generated raw outputs, processed result files, visualizations, and supporting documentation produced throughout the computational workflow, have been deposited in the open-access RepOD repository (https://doi.org/10.18150/HNUSRO).

## Abbreviations

The following abbreviations are used in this manuscript:

PTR	pterostilbene
AD	Alzheimer’s disease
HD	Huntington’s disease
PD	Parkinson’s disease
ALS	Amyotrophic Lateral Sclerosis
PPI network	protein–protein interaction (PPI) network
ALA	alanine
ARG	arginine
ASN	asparagine
ASP	aspartic acid
GLU	glutamic acid
GLY	glycine
HIS	histidine
ILE	isoleucine
LEU	leucine
LYS	lysine
MET	methionine
PHE	phenylalanine
SER	serine
SRC	C-SRC
THR	threonine
TRP	tryptophan
TYR	tyrosine
VAL	valine
