# Supplementary material for: Integrative Network Pharmacology and Molecular Docking Analysis Reveals the Multitarget Mechanisms of Pterostilbene in Neurodegenerative Diseases
Source: Pharmaceuticals (Basel). 2026 Jul 8;19(7):1053. doi: 10.3390/ph19071053 (PMC13415307; doi:10.3390/ph19071053)
Supplement: Supplementary file 1 [file pharmaceuticals-19-01053-s001.zip › pharmaceuticals-4355433-supplementary.pdf]

pharmaceuticals

MDPI

*Supplementary Materials*

# Integrative Network Pharmacology and Molecular Docking Analysis Reveals the Multitarget Mechanisms of Pterostilbene in Neurodegenerative Diseases

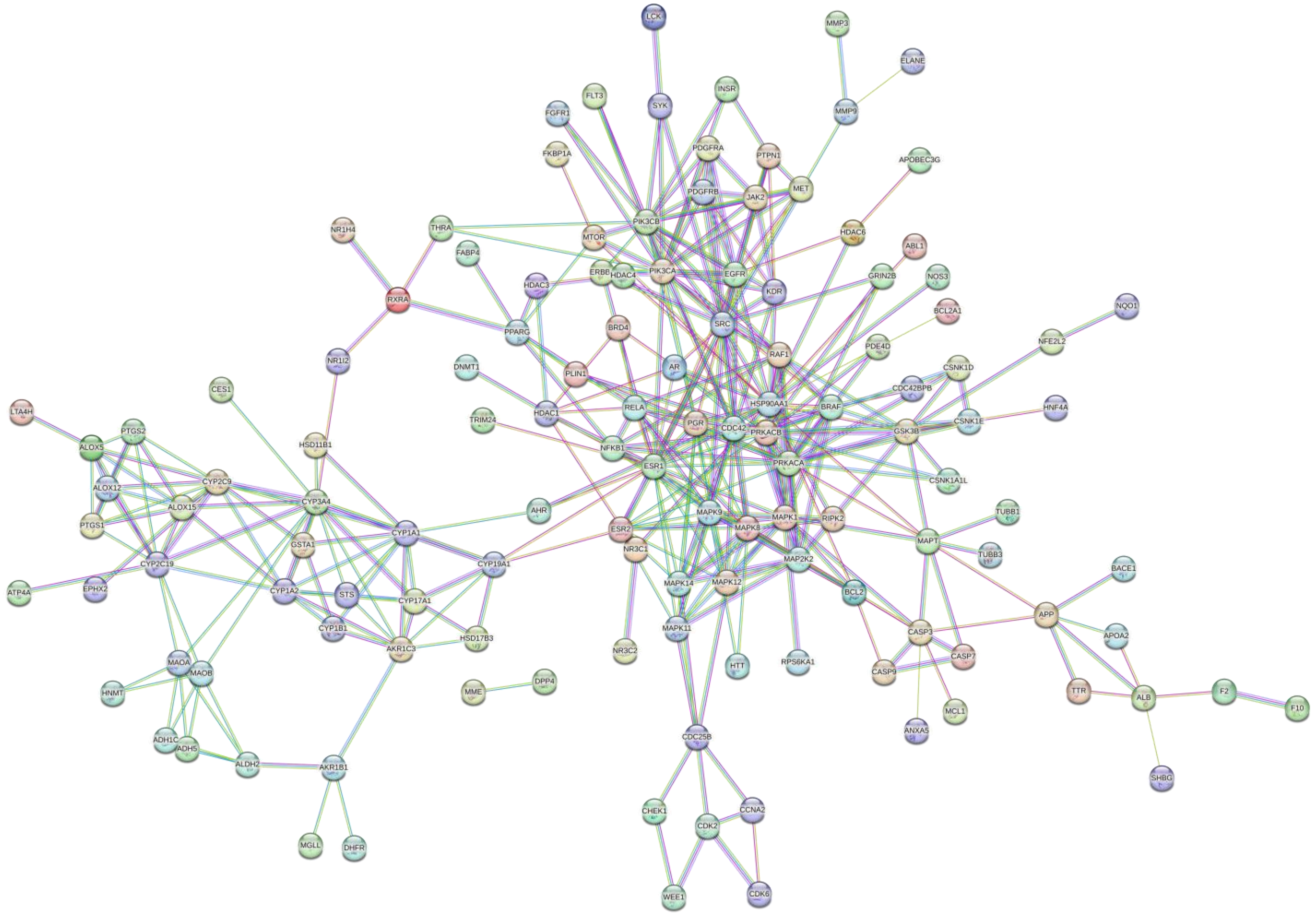


**Figure S1** PPI network analysis of PTR-AD: a giant network obtained after removing disconnected nodes. The figure is available as a high-resolution bitmap (.png format; "[giant network] PTR-AD in the online repository (https://doi.org/10.18150/HNUSRO) in PPI network_results.zip.

pharmaceuticals

MDPI

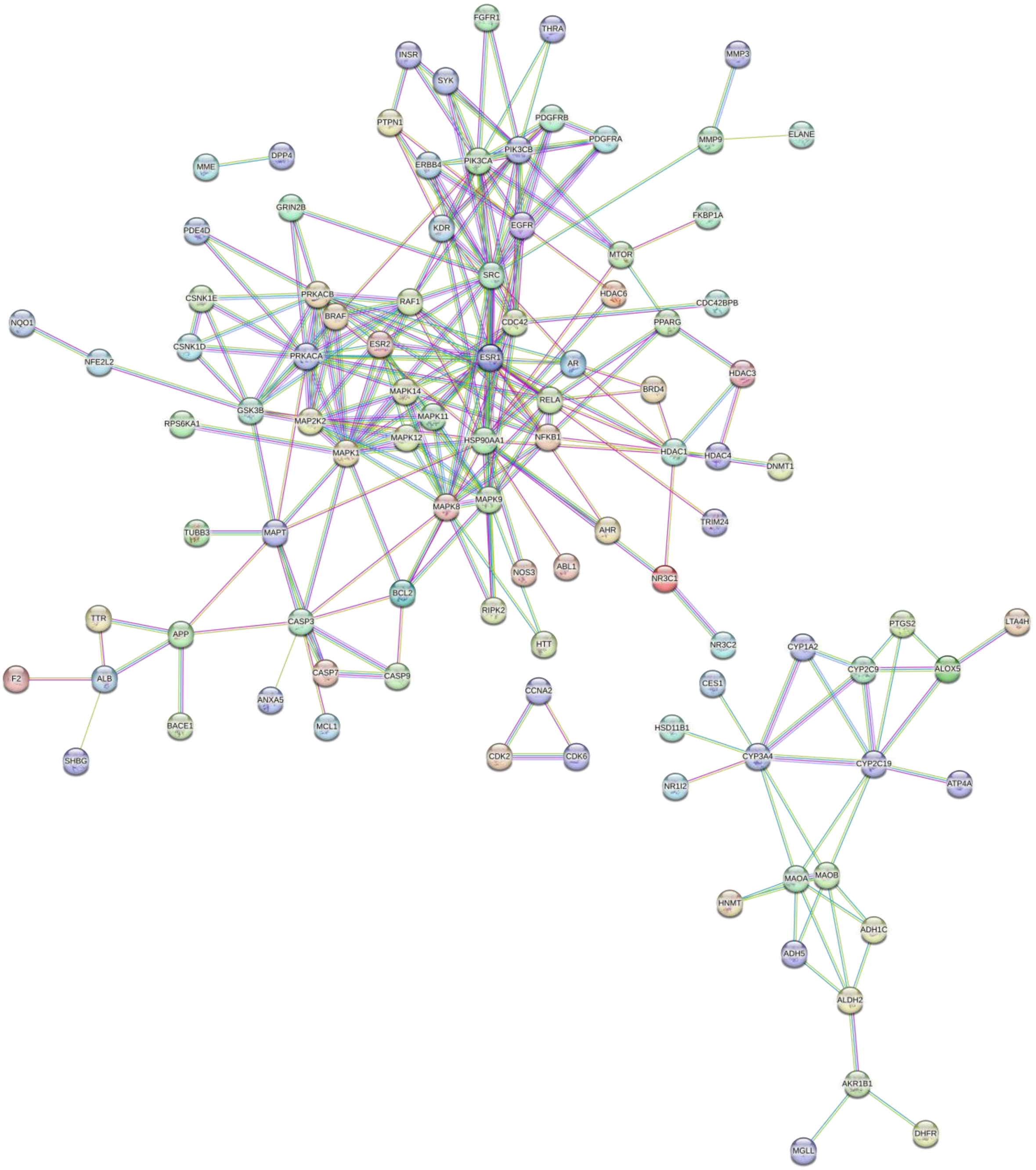


**Figure S2.** PPI network analysis of PTR-HD: a giant network obtained after removing disconnected nodes. The figure is available as a high-resolution bitmap (.png format; “[giant network] PTR-HD”) in the online repository (https://doi.org/10.18150/HNUSRO) in PPI network_results.zip.

pharmaceuticals

MDPI

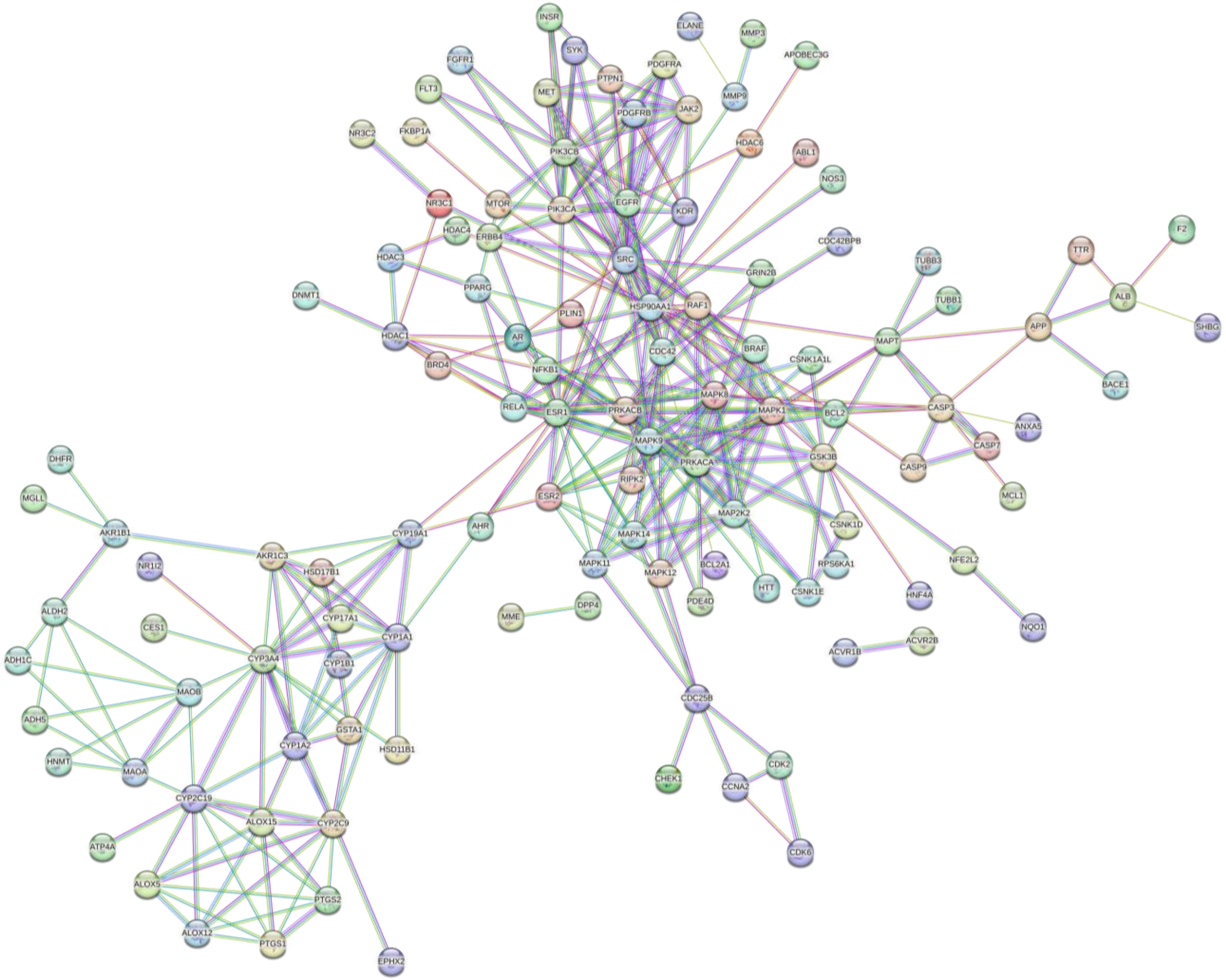


**Figure S3.** PPI network analysis of PTR-PD: a giant network obtained after removing disconnected nodes. The figure is available as a high-resolution bitmap (.png format; "[giant network] PTR-PD") in the online repository (https://doi.org/10.18150/HNUSRO) in PPI network_results.zip.

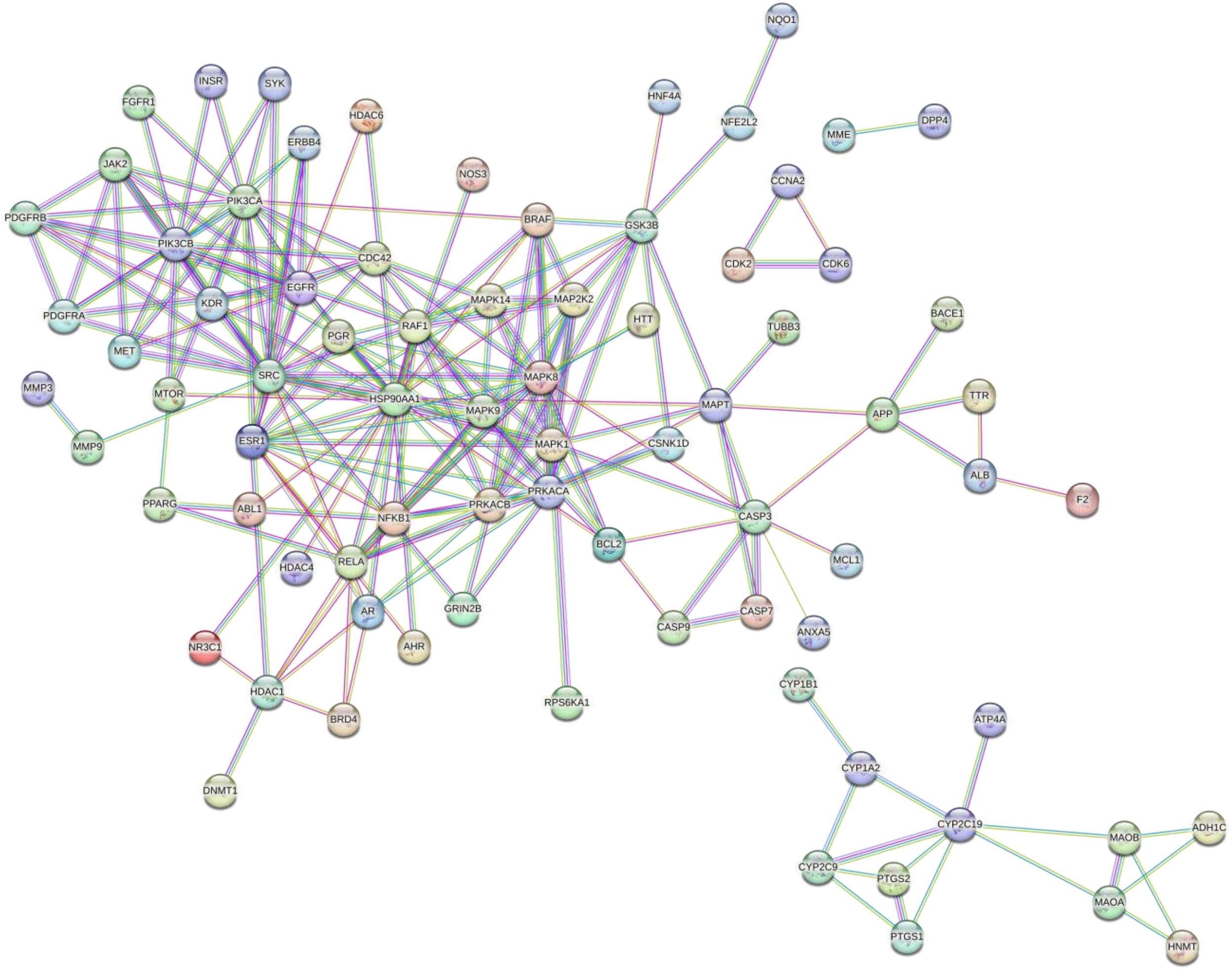


**Figure S4.** PPI network analysis of PTR-ALS: a giant network obtained after removing disconnected nodes. The figure is available as a high-resolution bitmap (.png format; "[giant network] PTR-ALS") in the online repository (https://doi.org/10.18150/HNUSRO) in PPI network_results.zip.

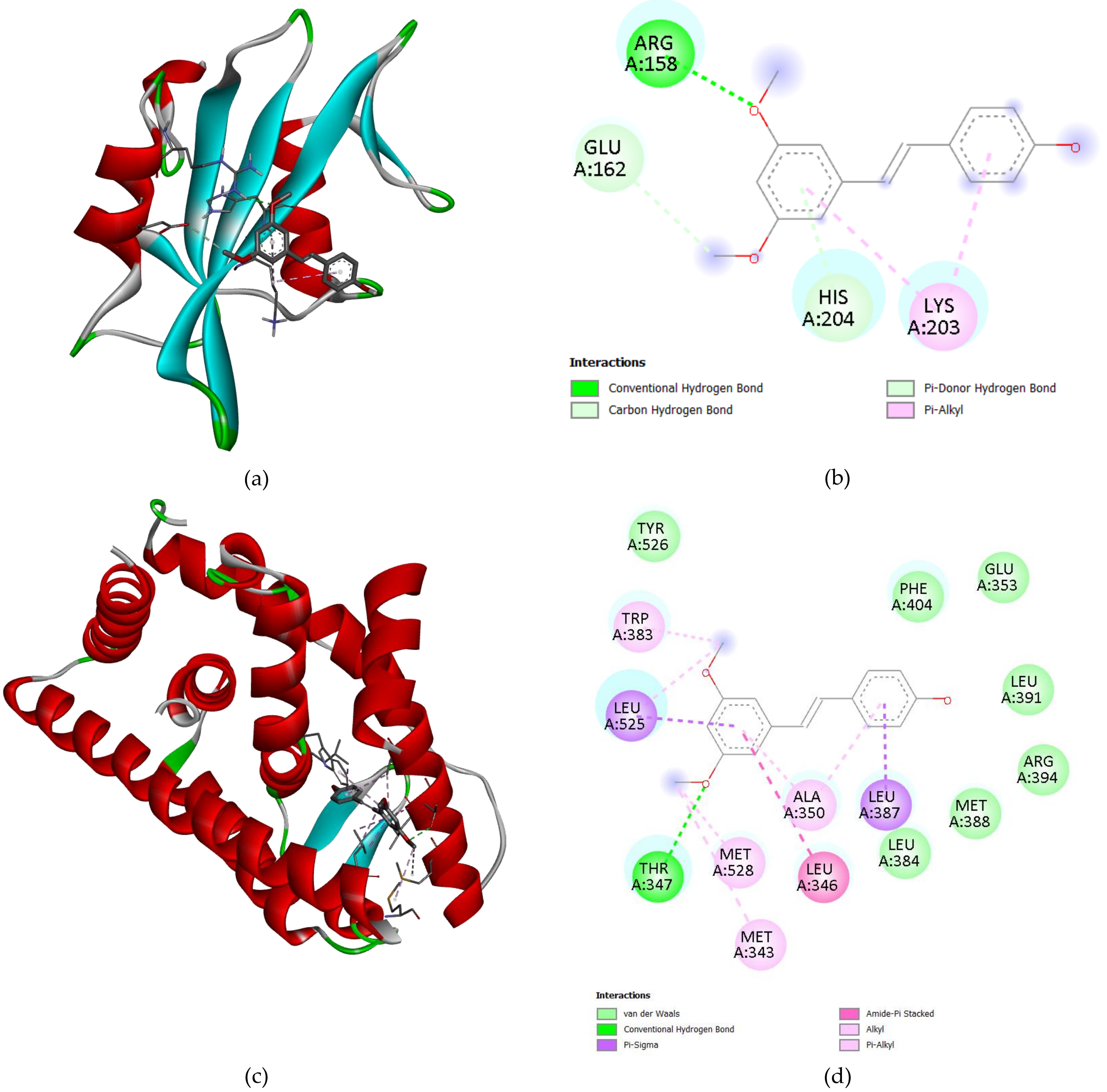
ARG
A:158
GLU
A:162
HIS
A:204
LYS
A:203
Interactions
Conventional Hydrogen Bond
Carbon Hydrogen Bond
Pi-Donor Hydrogen Bond
Pi-Alkyl
(a)
(b)
TYR
A:526
PHE
A:404
GLU
A:353
TRP
A:383
LEU
A:391
LEU
A:525
ARG
A:394
ALA
A:350
LEU
A:387
MET
A:388
LEU
A:384
THR
A:347
MET
A:528
LEU
A:346
MET
A:343
Interactions
van der Waals
Conventional Hydrogen Bond
Pi-Sigma
Amide-Pi Stacked
Alkyl
Pi-Alkyl
(c)
(d)

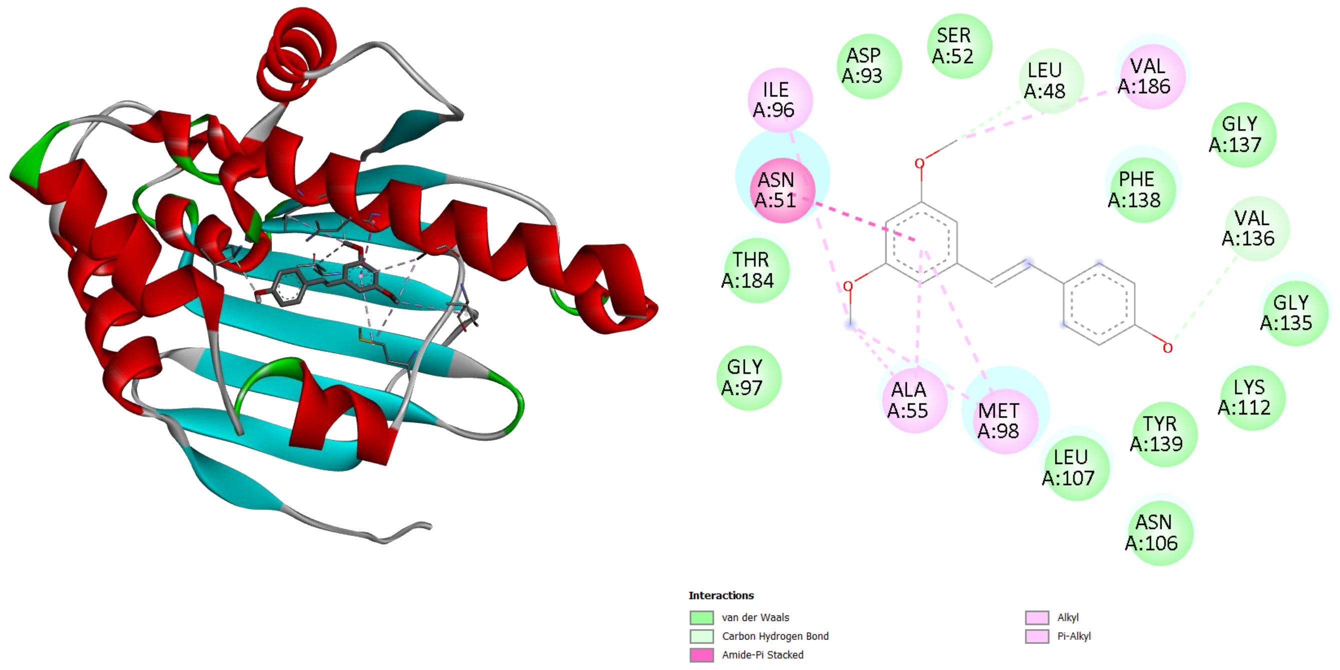


(e) (f)

**Figure S5.** Visualization of docking results of best ranked pose of key interactions in the binding pocket of SRC (PDB ID: 1A07, predicted binding energy of −4.8 kcal/mol), ESR1 (PDB ID: 1ERR, predicted binding energy of −7.2 kcal/mol), and HSP90AA1 (PDB ID: 1OSF, predicted binding energy of −6.9 kcal/mol) with pterostilbene (CID: 5281727). (a) 3D representation of PTR-SRC, (b) 2D representation of PTR-SRC, (c) 3D representation of PTR-ESR1, (d) 2D representation of PTR-ESR1, (e) 3D representation of PTR-HSP90AA1, (f) 2D representation of PTR-HSP90AA1. Legend: ALA – alanine, ARG – arginine, ASN – asparagine, ASP – aspartic acid, GLU – glutamic acid, GLY – glycine, HIS – histidine, ILE – isoleucine, LEU – leucine, LYS – lysine, MET – methionine, PHE – phenylalanine, SER – serine, THR – threonine, TRP – tryptophan, TYR – tyrosine, VAL – valine. Protein–ligand interactions are highlighted in green, whereas the cyan surface denotes the solvent-accessible surface area (SASA).

Molecular docking analysis demonstrated that PTR binds within the catalytic pocket of SRC kinase with a predicted binding energy of −4.8 kcal/mol (Figure S13a-b), indicating the weakest interaction among the analyzed targets. The ligand is stabilized by a conventional hydrogen bond with Arg158, which plays a key role in anchoring PTR in the active site. In addition, a weak carbon hydrogen bond with GLU162 contributes to maintaining the ligand orientation. The aromatic moieties of PTR engage in π-alkyl interactions with Lys203, providing hydrophobic stabilization of the complex. Furthermore, several residues, including HIS204, VAL202, TYR205, AND GLY239, form van der Waals contacts with PTR, enhancing steric complementarity and overall binding stability.

The strongest binding affinity was observed for estrogen receptor alpha (ESR1), with a predicted binding energy of −7.2 kcal/mol (Figure S13c-d). PTR was anchored within the ligand-binding domain through a conventional hydrogen bond with THR347, contributing to stabilization of its orientation in the binding pocket. The aromatic scaffold of PTR engaged in multiple hydrophobic interactions, including π–sigma and π–alkyl contacts with LEU346, LEU384, LEU387, LEU525, MET343, MET528, and ALA350, thereby enhancing complex stability. In addition, an amide–π stacked interaction with TRP383 further reinforced aromatic stacking interactions. Surrounding residues such as PHE404, TYR526, GLU353, ARG394, and LEU391 participated in van der Waals interactions, improving steric complementarity within the binding cavity. Overall, the progressively more favorable binding energies and increasingly extensive hydrophobic interaction networks indicate

that PTR adopts stable and energetically preferred conformations across the analyzed targets, with the highest predicted affinity toward ESR1.

For human heat shock protein 90 (Hsp90, HSP90AA1), PTR demonstrated an improved binding profile, with a predicted binding energy of −6.9 kcal/mol (Figure S13e-f). The ligand was accommodated within the binding pocket through a network of aromatic and hydrophobic interactions, including amide–π stacked contacts with Asn51 and π–alkyl interactions involving ALA55, MET98, and VAL186, which stabilized the aromatic scaffold of PTR. Additional hydrophobic contacts with ILE96 and LEU48 further supported ligand binding. Moreover, several residues, including THR184, SER52, ASP93, PHE138, TYR139, LEU107, ASN106, and GLY135-137, contributed van der Waals interactions, enhancing steric complementarity and overall complex stability.

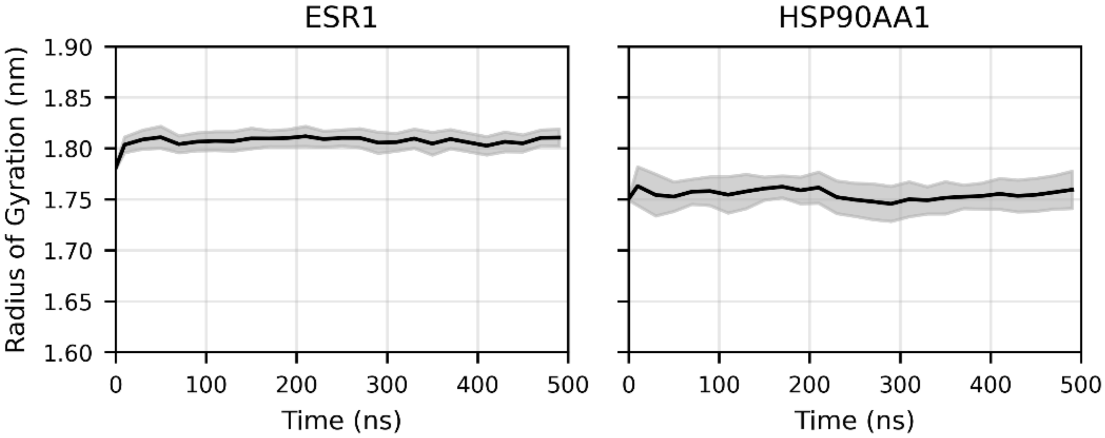


**Figure S6.** Radius of gyration (RG) of ESR1-PTR and HSP90AA1-PTR protein-ligand systems. Both RG analyses are presented as the average (black line) with the corresponding standard deviation (grey shaded area), calculated using 20 ns time windows.

**Table S1**. Potential molecular targets of pterostilbene (PTR). Target identifiers are provided according to the UniProtKB database (UniProt IDs). Yellow highlighted cells indicate duplicate targets between sets. SwissTarget, SuperPred, TargNet, PharmMapper - web-based target prediction tools. All data presented in this table are available as an .xlsx file ('Potential molecular targets of pterostilbene', sheet 'marked duplicates') in the online repository (https://doi.org/10.18150/HNUSRO).

| SwissTarget n=97 | SuperPred n=20 | TargNet n=68 | PharmMapper n=72 |
|---|---|---|---|
| O14757 | O15164 | O00748 | O14757 |
| O14976 | O75469 | O43353 | O76074 |
| O15379 | P02766 | O43570 | P00326 |
| O43353 | P05067 | O60240 | P00374 |
| O43570 | P09619 | O95977 | P00533 |
| O60674 | P09917 | P03372 | P00734 |
| O60885 | P10827 | P04054 | P00742 |
| P00519 | P18054 | P05067 | P00918 |
| P00533 | P19838 | P05177 | P02652 |
| P00915 | P20618 | P05186 | P02766 |
| P00918 | P27695 | P07949 | P02768 |
| P03372 | Q13526 | P08183 | P03372 |
| P04035 | Q13887 | P08842 | P04150 |
| P04049 | Q16236 | P09917 | P04179 |
| P04798 | Q16678 | P11511 | P04183 |
| P05067 | Q92731 | P14061 | P04278 |
| P05093 | Q99714 | P14174 | P04746 |
| P05177 | Q9BY41 | P14780 | P05091 |
| P06239 | Q9HAZ1 | P16050 | P06213 |
| P07451 | Q9Y345 | P16234 | P06401 |
| P08183 | | P18031 | P07900 |
| P08246 | | P20648 | P08235 |
| P08254 | | P21397 | P08246 |
| P08684 | | P21728 | P08263 |
| P09917 | | P22748 | P08473 |
| P10415 | | P23141 | P08581 |
| P10636 | | P23219 | P08758 |
| P11511 | | P23280 | P08842 |
| P11712 | | P24468 | P09960 |
| P14174 | | P26358 | P10275 |
| P14222 | | P27338 | P11309 |
| P15056 | | P30305 | P11310 |
| P16083 | | P31941 | P11362 |
| P17612 | | P33261 | P11766 |
| P21397 | | P35218 | P12931 |
| P22694 | | P35354 | P14061 |
| P22748 | | P35398 | P15090 |

| | | | |
|---|---|---|---|
| P23219 | | P35869 | P15121 |
| P23280 | | P37058 | P15559 |
| P23975 | | P37059 | P16083 |
| P24941 | | P41235 | P18031 |
| P28482 | | P42330 | P19793 |
| P28845 | | P43166 | P20248 |
| P30291 | | P51452 | P24941 |
| P33261 | | P55211 | P27487 |
| P33981 | | P56524 | P27707 |
| P34913 | | P60953 | P28845 |
| P35218 | | P63000 | P29373 |
| P35354 | | Q00975 | P29474 |
| P35869 | | Q00G26 | P35558 |
| P36507 | | Q04206 | P35968 |
| P36888 | | Q13224 | P37231 |
| P36896 | | Q14833 | P39900 |
| P42336 | | Q16548 | P42574 |
| P42338 | | Q16790 | P45452 |
| P42345 | | Q8N1Q1 | P49841 |
| P42685 | | Q92731 | P50135 |
| P42858 | | Q96RI1 | P52732 |
| P43166 | | Q99685 | P55210 |
| P43405 | | Q9BY41 | P56817 |
| P45983 | | Q9GZU7 | P62508 |
| P45984 | | Q9HC16 | P62942 |
| P47871 | | Q9HC97 | Q00534 |
| P48730 | | Q9NR96 | Q02127 |
| P49674 | | Q9UBN7 | Q04828 |
| P53778 | | Q9ULX7 | Q07343 |
| Q04206 | | Q9UNQ0 | Q08499 |
| Q07820 | | Q9Y2D0 | Q13126 |
| Q08345 | | | Q13370 |
| Q13509 | | | Q15303 |
| Q13547 | | | Q16539 |
| Q13627 | | | Q16836 |
| Q13705 | | | |
| Q13882 | | | |
| Q15418 | | | |
| Q15759 | | | |
| Q16539 | | | |
| Q16678 | | | |
| Q16790 | | | |
| Q16832 | | | |

| | | | |
|---|---|---|---|
| Q6DT37 | | | |
| Q8N1Q1 | | | |
| Q8N752 | | | |
| Q96D53 | | | |
| Q9H2G2 | | | |
| Q9H4B7 | | | |
| Q9NRM7 | | | |
| Q9NRP7 | | | |
| Q9NY57 | | | |
| Q9UBE8 | | | |
| Q9UBN7 | | | |
| Q9UF33 | | | |
| Q9UK32 | | | |
| Q9UKE5 | | | |
| Q9ULX7 | | | |
| Q9Y2D0 | | | |
| Q9Y5S2 | | | |

**Table S2.** List of 215 unique PTR targets. Target identifiers are provided according to the UniProtKB database (UniProt IDs). SwissTarget, SuperPred, TargNet, PharmMapper - web-based target prediction tools. All data presented in this table are available as an .xlsx file ('Potential molecular targets of pterostilbene', sheet 'no duplicates') in the online repository (https://doi.org/10.18150/HNUSRO).

| SwissTarget (n=97) | SuperPred (n=14) | TargNet (n=41) | PharmMapper (n=63) |
|---|---|---|---|
| O14757 | O15164 | O00748 | O76074 |
| O14976 | O75469 | O60240 | P00326 |
| O15379 | P09619 | O95977 | P00374 |
| O43353 | P10827 | P04054 | P00734 |
| O43570 | P18054 | P05186 | P00742 |
| O60674 | P19838 | P07949 | P02652 |
| O60885 | P20618 | P14780 | P02766 |
| P00519 | P27695 | P16050 | P02768 |
| P00533 | Q13526 | P16234 | P04150 |
| P00915 | Q13887 | P20648 | P04179 |
| P00918 | Q16236 | P21728 | P04183 |
| P03372 | Q99714 | P23141 | P04278 |
| P04035 | Q9HAZ1 | P24468 | P04746 |
| P04049 | Q9Y345 | P26358 | P05091 |
| P04798 | | P27338 | P06213 |
| P05067 | | P30305 | P06401 |
| P05093 | | P31941 | P07900 |
| P05177 | | P35398 | P08235 |
| P06239 | | P37058 | P08263 |
| P07451 | | P37059 | P08473 |
| P08183 | | P41235 | P08581 |
| P08246 | | P42330 | P08758 |
| P08254 | | P51452 | P08842 |
| P08684 | | P55211 | P09960 |
| P09917 | | P56524 | P10275 |
| P10415 | | P60953 | P11309 |
| P10636 | | P63000 | P11310 |
| P11511 | | Q00975 | P11362 |
| P11712 | | Q00G26 | P11766 |
| P14174 | | Q13224 | P12931 |
| P14222 | | Q14833 | P14061 |
| P15056 | | Q16548 | P15090 |
| P16083 | | Q92731 | P15121 |
| P17612 | | Q96RI1 | P15559 |
| P21397 | | Q99685 | P18031 |
| P22694 | | Q9BY41 | P19793 |
| P22748 | | Q9GZU7 | P20248 |

| | | | |
|---|---|---|---|
| P23219 | | Q9HC16 | P27487 |
| P23280 | | Q9HC97 | P27707 |
| P23975 | | Q9NR96 | P29373 |
| P24941 | | Q9UNQ0 | P29474 |
| P28482 | | | P35558 |
| P28845 | | | P35968 |
| P30291 | | | P37231 |
| P33261 | | | P39900 |
| P33981 | | | P42574 |
| P34913 | | | P45452 |
| P35218 | | | P49841 |
| P35354 | | | P50135 |
| P35869 | | | P52732 |
| P36507 | | | P55210 |
| P36888 | | | P56817 |
| P36896 | | | P62508 |
| P42336 | | | P62942 |
| P42338 | | | Q00534 |
| P42345 | | | Q02127 |
| P42685 | | | Q04828 |
| P42858 | | | Q07343 |
| P43166 | | | Q08499 |
| P43405 | | | Q13126 |
| P45983 | | | Q13370 |
| P45984 | | | Q15303 |
| P47871 | | | Q16836 |
| P48730 | | | |
| P49674 | | | |
| P53778 | | | |
| Q04206 | | | |
| Q07820 | | | |
| Q08345 | | | |
| Q13509 | | | |
| Q13547 | | | |
| Q13627 | | | |
| Q13705 | | | |
| Q13882 | | | |
| Q15418 | | | |
| Q15759 | | | |
| Q16539 | | | |
| Q16678 | | | |
| Q16790 | | | |
| Q16832 | | | |

| | | | |
|---|---|---|---|
| Q6DT37 | | | |
| Q8N1Q1 | | | |
| Q8N752 | | | |
| Q96D53 | | | |
| Q9H2G2 | | | |
| Q9H4B7 | | | |
| Q9NRM7 | | | |
| Q9NRP7 | | | |
| Q9NY57 | | | |
| Q9UBE8 | | | |
| Q9UBN7 | | | |
| Q9UF33 | | | |
| Q9UK32 | | | |
| Q9UKE5 | | | |
| Q9ULX7 | | | |
| Q9Y2D0 | | | |
| Q9Y5S2 | | | |

**Table S3.** Shared targets of PTR and neurodegenerative diseases identified by Venn diagram analysis. Target identifiers are provided according to the UniProtKB database (UniProt IDs). Legend: PTR – pterostilbene, AD – Alzheimer's disease, HD – Huntington's disease, PD – Parkinson's disease, ALS – amyotrophic lateral sclerosis. All data presented in this table are available as an .xlsx file ('Venn diagram_intersection', sheet 'summary') in the online repository (https://doi.org/10.18150/HNUSRO).

| PTR-AD (n=181) | PTR-HD (n=128) | PTR-PD (n=165) | PTR-ALS (n=109) |
|---|---|---|---|
| P19793 | P04150 | P04150 | P04150 |
| P04150 | Q9UBN7 | Q9UBN7 | Q9UBN7 |
| Q9UBN7 | P07949 | P07949 | P07949 |
| P07949 | P35354 | P09917 | P35354 |
| P09917 | P09917 | O14757 | O14757 |
| O14757 | Q13224 | P10415 | Q13224 |
| P10415 | P10415 | P10275 | P10415 |
| P10275 | P10275 | O15379 | P10275 |
| P06239 | P03372 | P52732 | P03372 |
| O15379 | P00533 | Q16548 | P00533 |
| P52732 | O15379 | O60240 | P52732 |
| Q16548 | P52732 | Q9NR96 | Q9NR96 |
| O60240 | P00734 | P45983 | P00734 |
| Q9NR96 | P00918 | Q92731 | P00918 |
| P45983 | P45983 | P55210 | P45983 |
| Q92731 | Q9HC97 | P00519 | P55210 |
| P55210 | Q92731 | Q07343 | P00519 |
| P00519 | P55210 | P22694 | P29474 |
| Q07343 | P00519 | P04035 | P19838 |
| P09960 | P29474 | O60885 | P11310 |
| P22694 | P19838 | Q16832 | P24941 |
| P04035 | P11310 | P28482 | P15056 |
| O60885 | Q07343 | P02766 | P22694 |
| Q16832 | O14976 | P18031 | P04035 |
| P28482 | P24941 | O43353 | O60885 |
| P02766 | P09960 | P53778 | Q99714 |
| P18031 | P15056 | P14061 | P50135 |
| O43353 | P22694 | P04049 | Q96D53 |
| P53778 | P04035 | P20618 | P28482 |
| P04049 | O60885 | Q96RI1 | P35869 |
| P06401 | Q99714 | P55211 | P02766 |
| P20618 | P50135 | P42345 | P63000 |
| Q96RI1 | P28482 | P05067 | P36507 |
| P55211 | P35869 | P42336 | P00374 |
| P42345 | P02766 | O60674 | P00326 |
| P05067 | P18031 | Q08345 | Q16539 |
| P42336 | P05091 | P42574 | P26358 |
| O60674 | P63000 | P08263 | P42858 |
| Q08345 | P36507 | P11712 | P04049 |
| P42574 | P00374 | P42330 | P06401 |

| | | | |
|---|---|---|---|
| P08263 | P00326 | P62942 | P20618 |
| P11712 | Q16539 | P35398 | P60953 |
| P42330 | P26358 | P49841 | P56817 |
| P62942 | O43353 | P28845 | Q04206 |
| P35398 | P53778 | P23219 | Q9BY41 |
| P49841 | P42858 | P27695 | P27338 |
| P28845 | P04049 | Q13627 | P45984 |
| P23219 | P20618 | P16083 | P55211 |
| P27695 | P60953 | P08183 | P37231 |
| Q13627 | P56817 | P16234 | P42345 |
| P16083 | Q04206 | P08235 | Q9Y345 |
| P08183 | P15121 | P08581 | P05067 |
| P16234 | Q9BY41 | P08473 | P42336 |
| P00915 | P49674 | P48730 | Q13509 |
| P08235 | P27338 | P05093 | Q15418 |
| P08581 | P45984 | P11309 | P07900 |
| P08473 | P55211 | P22748 | O60674 |
| P48730 | P37231 | P16050 | P11362 |
| P05093 | P42345 | Q13705 | P14780 |
| P37058 | Q9Y345 | Q16236 | P21397 |
| P11309 | P05067 | Q07820 | Q13126 |
| P22748 | P42336 | P36888 | P42574 |
| P16050 | Q13509 | Q15303 | P14222 |
| Q16236 | Q15418 | P02768 | P11712 |
| Q07820 | P07900 | P23141 | P12931 |
| Q9H2G2 | P23975 | Q08499 | Q13526 |
| P36888 | Q15759 | Q9UNQ0 | P09619 |
| Q15303 | P11362 | Q16836 | Q00975 |
| P02768 | P14780 | P08684 | P49841 |
| P23141 | P21397 | P42338 | Q16678 |
| Q08499 | Q13126 | Q14833 | Q9NRP7 |
| Q9UNQ0 | P42574 | P17612 | P23219 |
| Q16836 | P14222 | P27487 | P27695 |
| P08684 | P11712 | P08254 | P14174 |
| P42338 | P12931 | Q99685 | Q13547 |
| P17612 | Q13526 | P10636 | Q13627 |
| P27487 | P62942 | P20648 | P08183 |
| P08254 | P09619 | P06213 | P16234 |
| Q99685 | Q00975 | P56524 | P08581 |
| P10827 | P49841 | P11766 | P08473 |
| P10636 | P21728 | P35354 | P48730 |
| P00742 | P28845 | O43570 | P04179 |
| P20648 | P08246 | Q13224 | P22748 |
| P06213 | Q9Y5S2 | Q9HC16 | Q13705 |
| P56524 | P27695 | P35558 | Q16236 |
| O15164 | P14174 | P03372 | Q07820 |

| | | | |
|---|---|---|---|
| P11766 | Q13547 | P00533 | P35968 |
| P35354 | Q13627 | P00734 | Q02127 |
| O43570 | P08183 | P00918 | Q15303 |
| Q13224 | P16234 | Q9H4B7 | P41235 |
| Q9HC16 | P08235 | P29474 | P02768 |
| P35558 | P08473 | P19838 | Q9UNQ0 |
| P03372 | P48730 | P11310 | P15559 |
| P00533 | P04179 | Q8N752 | Q16836 |
| Q13887 | O75469 | O14976 | P08758 |
| P00734 | P22748 | P24941 | P42338 |
| P00918 | Q16236 | P15056 | P43405 |
| Q9HC97 | Q07820 | Q99714 | P17612 |
| Q9H4B7 | Q9H2G2 | P50135 | P27487 |
| P29474 | P35968 | Q96D53 | P05177 |
| P19838 | Q15303 | P35869 | Q00534 |
| P11310 | P02768 | P05091 | P20248 |
| P30291 | P23141 | P63000 | P08254 |
| P15090 | O76074 | P36507 | P10636 |
| Q8N752 | Q08499 | P00374 | P39900 |
| O14976 | Q9UNQ0 | P00326 | P20648 |
| P24941 | P15559 | Q16539 | P33261 |
| P15056 | Q16836 | P26358 | P06213 |
| Q99714 | P08684 | P42858 | P56524 |
| P50135 | P08758 | P60953 | |
| Q96D53 | P42338 | P56817 | |
| P04746 | P43405 | Q04206 | |
| P35869 | P17612 | P15121 | |
| P05091 | P27487 | P49674 | |
| P63000 | P05177 | P27338 | |
| P36507 | P04278 | P45984 | |
| P00374 | Q00534 | P37231 | |
| P00326 | P20248 | Q9Y345 | |
| Q16539 | P08254 | Q13509 | |
| P26358 | Q99685 | Q15418 | |
| P42858 | P10827 | P07900 | |
| P60953 | P10636 | P23975 | |
| P56817 | P20648 | P18054 | |
| Q04206 | P33261 | Q15759 | |
| P15121 | P06213 | P11362 | |
| Q9BY41 | P56524 | P14780 | |
| P49674 | O15164 | P21397 | |
| P27338 | P11766 | P14222 | |
| P45984 | | P05186 | |
| P02652 | | P11511 | |
| P37231 | | P12931 | |
| Q9Y345 | | Q13526 | |

| | | | |
|---|---|---|---|
| Q13509 | | P09619 | |
| Q15418 | | Q00975 | |
| P07900 | | Q16790 | |
| P23975 | | Q16678 | |
| P18054 | | P21728 | |
| Q15759 | | P08246 | |
| P11362 | | P35218 | |
| P14780 | | Q9Y5S2 | |
| P21397 | | P36896 | |
| Q13126 | | P14174 | |
| P14222 | | Q9NY57 | |
| P05186 | | Q13547 | |
| P11511 | | P04798 | |
| P12931 | | P04179 | |
| Q13526 | | O75469 | |
| P09619 | | P35968 | |
| Q00975 | | P30305 | |
| Q16790 | | Q02127 | |
| Q16678 | | P41235 | |
| P21728 | | O76074 | |
| P08246 | | Q13370 | |
| P35218 | | P15559 | |
| Q9Y5S2 | | P08758 | |
| P14174 | | P43405 | |
| Q9NY57 | | P34913 | |
| Q13547 | | P05177 | |
| P42685 | | P04278 | |
| P04798 | | Q00534 | |
| P04179 | | P20248 | |
| O75469 | | P24468 | |
| P35968 | | P45452 | |
| P30305 | | P39900 | |
| Q02127 | | P33261 | |
| P41235 | | | |
| O76074 | | | |
| Q13370 | | | |
| P15559 | | | |
| P08758 | | | |
| P43405 | | | |
| P34913 | | | |
| P05177 | | | |
| P04278 | | | |
| Q00534 | | | |
| P20248 | | | |
| P24468 | | | |
| P45452 | | | |

| | | | |
|---|---|---|---|
| P39900 | | | |
| P33261 | | | |
| P08842 | | | |

pharmaceuticals MDPI

**Table S4** Redundancy-reduced GO Biological Process enrichment results for PTR-AD obtained using REVIGO semantic clustering. Legend: In highlighted REVIGO clusters, representative terms are shown in bold, whereas semantically related terms assigned to the same representative cluster are indicated using the same background color. TermID denotes the GO identifier, Name indicates the biological process, and Value corresponds to the input statistical value used for REVIGO analysis. LogSize and Frequency describe the relative size and occurrence frequency of each GO term in the Gene Ontology database, whereas Uniqueness and Dispensability indicate semantic distinctiveness and redundancy, respectively. Lower dispensability values indicate less redundant and more representative GO terms.

| TermID | Name | Value | LogSize | Frequency | Uniqueness | Dispensability |
|---|---|---|---|---|---|---|
| GO:0000165 | MAPK cascade | -8.40 | 2.35 | 1.26 | 0.87 | 0.228 |
| GO:0001819 | positive regulation of cytokine production | -6.44 | 2.70 | 2.81 | 0.90 | 0.430 |
| GO:0002376 | immune system process | -5.87 | 3.39 | 13.66 | 1.00 | 0.000 |
| GO:0002682 | regulation of immune system process | -8.21 | 3.22 | 9.29 | 0.96 | 0.048 |
| GO:0002764 | immune response-regulating signaling pathway | -5.59 | 2.62 | 2.33 | 0.79 | 0.318 |
| GO:0006629 | lipid metabolic process | -13.60 | 3.09 | 6.85 | 0.97 | 0.172 |
| **GO:0006690** | **icosanoid metabolic process** | -12.48 | 2.07 | 0.65 | 0.94 | 0.104 |
| GO:0006631 | fatty acid metabolic process | 6631.00 | -11.82 | 2.52 | 1.85 | 0.908 |
| GO:0046456 | icosanoid biosynthetic process | 46456.00 | -5.68 | 1.72 | 0.29 | 0.947 |
| GO:0006954 | inflammatory response | -6.98 | 2.78 | 3.37 | 0.89 | 0.125 |
| GO:0007169 | cell surface receptor protein tyrosine kinase signaling pathway | -14.58 | 2.65 | 2.48 | 0.85 | 0.120 |
| GO:0007611 | learning or memory | -6.34 | 2.46 | 1.61 | 1.00 | 0.000 |
| GO:0008202 | steroid metabolic process | -12.82 | 2.41 | 1.45 | 0.95 | 0.000 |
| GO:0008210 | estrogen metabolic process | -6.32 | 1.60 | 0.22 | 0.93 | 0.482 |
| GO:0009410 | response to xenobiotic stimulus | -17.37 | 2.63 | 2.37 | 0.79 | 0.363 |
| GO:0009628 | response to abiotic stimulus | -14.91 | 3.06 | 6.41 | 0.90 | 0.139 |
| GO:0009636 | response to toxic substance | -10.86 | 2.41 | 1.43 | 0.80 | 0.338 |
| **GO:0009725** | **response to hormone** | -20.76 | 2.92 | 4.65 | 0.70 | 0.000 |
| GO:0032870 | cellular response to hormone stimulus | 32870.00 | -13.97 | 2.75 | 3.16 | 0.669 |
| GO:0048545 | response to steroid hormone | 48545.00 | -11.06 | 2.45 | 1.58 | 0.684 |
| GO:0070848 | response to growth factor | 70848.00 | -9.67 | 2.72 | 2.92 | 0.801 |
| GO:0010038 | response to metal ion | -10.89 | 2.54 | 1.95 | 0.79 | 0.353 |
| GO:0010506 | regulation of autophagy | -5.74 | 2.58 | 2.11 | 0.93 | 0.174 |

| TermID | Name | Value | LogSize | Frequency | Uniqueness | Dispensability |
|---|---|---|---|---|---|---|
| GO:0012501 | programmed cell death | -6.97 | 3.05 | 6.34 | 1.00 | 0.005 |
| GO:0019216 | regulation of lipid metabolic process | -10.41 | 2.53 | 1.88 | 0.93 | 0.044 |
| GO:0034097 | response to cytokine | -6.33 | 2.93 | 4.77 | 0.77 | 0.404 |
| **GO:0034614** | **cellular response to reactive oxygen species** | -13.30 | 2.10 | 0.70 | 0.73 | 0.462 |
| GO:0042542 | response to hydrogen peroxide | 42542.00 | -7.41 | 2.00 | 0.56 | 0.770 |
| **GO:0034762** | **regulation of transmembrane transport** | -5.74 | 2.60 | 2.25 | 0.94 | 0.045 |
| GO:0043269 | regulation of monoatomic ion transport | 43269.00 | -5.68 | 2.61 | 2.30 | 0.949 |
| **GO:0043408** | **regulation of MAPK cascade** | -12.12 | 2.81 | 3.66 | 0.87 | 0.000 |
| GO:0070372 | regulation of ERK1 and ERK2 cascade | 70372.00 | -6.55 | 2.45 | 1.57 | 0.878 |
| GO:0050865 | regulation of cell activation | -6.73 | 2.81 | 3.62 | 0.95 | 0.048 |
| GO:0051897 | positive regulation of phosphatidylinositol 3-kinase/protein kinase B signal transduction | -7.01 | 2.30 | 1.11 | 0.87 | 0.495 |
| **GO:0071396** | **cellular response to lipid** | -18.00 | 2.76 | 3.19 | 0.68 | 0.379 |
| GO:0031960 | response to corticosteroid | 31960.00 | -8.08 | 2.20 | 0.88 | 0.687 |
| GO:0032355 | response to estradiol | 32355.00 | -6.73 | 2.06 | 0.64 | 0.731 |
| GO:0032496 | response to lipopolysaccharide | 32496.00 | -10.32 | 2.50 | 1.78 | 0.703 |
| GO:0043401 | steroid hormone receptor signaling pathway | 43401.00 | -6.45 | 1.90 | 0.44 | 0.661 |
| GO:0062197 | cellular response to chemical stress | 62197.00 | -15.27 | 2.45 | 1.59 | 0.749 |
| GO:0071383 | cellular response to steroid hormone stimulus | 71383.00 | -8.59 | 2.19 | 0.87 | 0.668 |
| GO:0071466 | cellular response to xenobiotic stimulus | -7.65 | 2.29 | 1.10 | 0.77 | 0.488 |
| **GO:0071900** | **regulation of protein serine/threonine kinase activity** | -9.55 | 2.07 | 0.65 | 0.88 | 0.197 |
| GO:0030162 | regulation of proteolysis | 30162.00 | -7.01 | 2.59 | 2.16 | 0.902 |
| GO:0043405 | regulation of MAP kinase activity | 43405.00 | -8.46 | 1.81 | 0.36 | 0.888 |
| GO:0090218 | positive regulation of lipid kinase activity | 90218.00 | -6.32 | 0.30 | 0.01 | 0.914 |
| **GO:0080135** | **regulation of cellular response to stress** | -9.30 | 2.73 | 3.01 | 0.87 | 0.329 |
| GO:0050727 | regulation of inflammatory response | 50727.00 | -9.06 | 2.63 | 2.38 | 0.880 |
| GO:0098754 | detoxification | -6.37 | 2.18 | 0.84 | 0.81 | 0.315 |
| GO:1900407 | regulation of cellular response to oxidative stress | -7.18 | 1.34 | 0.12 | 0.91 | 0.450 |
| GO:1904645 | response to amyloid-beta | -5.68 | 1.74 | 0.30 | 0.80 | 0.279 |
| GO:2000377 | regulation of reactive oxygen species metabolic process | -9.92 | 2.15 | 0.79 | 0.94 | 0.155 |
| GO:2000379 | positive regulation of reactive oxygen species metabolic process | -6.83 | 1.83 | 0.38 | 0.93 | 0.143 |

| TermID | Name | Value | LogSize | Frequency | Uniqueness | Dispensability |
|---|---|---|---|---|---|---|
| **GO:2001233** | **regulation of apoptotic signaling pathway** | -9.18 | 2.58 | 2.15 | 0.86 | 0.402 |
| GO:0043069 | negative regulation of programmed cell death | 43069.00 | -14.89 | 2.97 | 5.23 | 0.920 |
| GO:2001243 | negative regulation of intrinsic apoptotic signaling pathway | 2001243.00 | -6.48 | 2.04 | 0.61 | 0.865 |

**Table S5.** Redundancy-reduced GO Biological Process enrichment results for PTR-HD obtained using REVIGO semantic clustering. Legend: In highlighted REVIGO clusters, representative terms are shown in bold, whereas semantically related terms assigned to the same representative cluster are indicated using the same background color. TermID denotes the GO identifier, Name indicates the biological process, and Value corresponds to the input statistical value used for REVIGO analysis. LogSize and Frequency describe the relative size and occurrence frequency of each GO term in the Gene Ontology database, whereas Uniqueness and Dispensability indicate semantic distinctiveness and redundancy, respectively. Lower dispensability values indicate less redundant and more representative GO terms.

| TermID | Name | Value | LogSize | Frequency | Uniqueness | Dispensability |
|---|---|---|---|---|---|---|
| GO:0000165 | MAPK cascade | -10.61 | 2.35 | 1.26 | 0.86 | 0.228 |
| GO:0001775 | cell activation | -5.74 | 2.89 | 4.35 | 0.99 | 0.176 |
| GO:0002376 | immune system process | -6.50 | 3.39 | 13.66 | 1.00 | 0.000 |
| GO:0002682 | regulation of immune system process | -6.70 | 3.22 | 9.29 | 0.96 | 0.048 |
| GO:0002768 | immune response-regulating cell surface receptor signaling pathway | -5.55 | 2.46 | 1.62 | 0.77 | 0.472 |
| GO:0006629 | lipid metabolic process | -7.14 | 3.09 | 6.85 | 0.97 | 0.178 |
| **GO:0006631** | **fatty acid metabolic process** | -7.30 | 2.52 | 1.85 | 0.94 | 0.000 |
| GO:0006690 | icosanoid metabolic process | 6690.00 | -6.37 | 2.07 | 0.65 | 0.960 |
| GO:0008202 | steroid metabolic process | 8202.00 | -6.74 | 2.41 | 1.45 | 0.959 |
| GO:0006954 | inflammatory response | -5.25 | 2.78 | 3.37 | 0.88 | 0.365 |
| GO:0007169 | cell surface receptor protein tyrosine kinase signaling pathway | -11.75 | 2.65 | 2.48 | 0.86 | 0.120 |
| GO:0007611 | learning or memory | -8.32 | 2.46 | 1.61 | 0.99 | 0.000 |
| GO:0009410 | response to xenobiotic stimulus | -13.70 | 2.63 | 2.37 | 0.84 | 0.363 |
| GO:0009611 | response to wounding | -5.59 | 2.66 | 2.57 | 0.88 | 0.120 |
| GO:0009636 | response to toxic substance | -7.34 | 2.41 | 1.43 | 0.85 | 0.338 |
| **GO:0009725** | **response to hormone** | -15.82 | 2.92 | 4.65 | 0.79 | 0.000 |
| GO:0032870 | cellular response to hormone stimulus | 32870.00 | -8.89 | 2.75 | 3.16 | 0.765 |
| GO:0070848 | response to growth factor | 70848.00 | -11.24 | 2.72 | 2.92 | 0.880 |
| GO:0010038 | response to metal ion | -9.29 | 2.54 | 1.95 | 0.84 | 0.353 |
| GO:0010506 | regulation of autophagy | -6.12 | 2.58 | 2.11 | 0.94 | 0.177 |
| GO:0010817 | regulation of hormone levels | -5.39 | 2.76 | 3.20 | 0.97 | 0.034 |
| GO:0012501 | programmed cell death | -7.50 | 3.05 | 6.34 | 1.00 | 0.005 |
| GO:0030162 | regulation of proteolysis | -8.77 | 2.59 | 2.16 | 0.91 | 0.157 |
| GO:0030522 | intracellular receptor signaling pathway | -7.39 | 2.31 | 1.15 | 0.87 | 0.473 |
| GO:0031346 | positive regulation of cell projection organization | -5.32 | 2.55 | 1.99 | 0.94 | 0.160 |

| TermID | Name | Value | LogSize | Frequency | Uniqueness | Dispensability |
|---|---|---|---|---|---|---|
| GO:0033554 | cellular response to stress | -14.14 | 3.22 | 9.34 | 0.84 | 0.446 |
| GO:0033993 | response to lipid | -15.69 | 2.95 | 4.99 | 0.82 | 0.407 |
| **GO:0034614** | **cellular response to reactive oxygen species** | -13.18 | 2.10 | 0.70 | 0.78 | 0.472 |
| GO:0042542 | response to hydrogen peroxide | 42542.00 | -7.81 | 2.00 | 0.56 | 0.810 |
| GO:0038083 | peptidyl-tyrosine autophosphorylation | -6.30 | 0.95 | 0.04 | 0.98 | 0.102 |
| **GO:0043269** | **regulation of monoatomic ion transport** | -7.75 | 2.61 | 2.30 | 0.93 | 0.038 |
| GO:0034762 | regulation of transmembrane transport | 34762.00 | -6.82 | 2.60 | 2.25 | 0.924 |
| GO:0051924 | regulation of calcium ion transport | 51924.00 | -5.74 | 2.34 | 1.24 | 0.932 |
| **GO:0043406** | **positive regulation of MAP kinase activity** | -7.33 | 1.57 | 0.20 | 0.78 | 0.457 |
| GO:0045862 | positive regulation of proteolysis | 45862.00 | -5.71 | 2.32 | 1.16 | 0.896 |
| GO:0070372 | regulation of ERK1 and ERK2 cascade | 70372.00 | -6.03 | 2.45 | 1.57 | 0.823 |
| GO:0090218 | positive regulation of lipid kinase activity | 90218.00 | -5.99 | 0.30 | 0.01 | 0.930 |
| **GO:0043408** | **regulation of MAPK cascade** | -12.09 | 2.81 | 3.66 | 0.82 | 0.000 |
| GO:0051896 | regulation of phosphatidylinositol 3-kinase/protein kinase B signal transduction | 51896.00 | -5.41 | 2.44 | 1.56 | 0.831 |
| GO:0051897 | positive regulation of phosphatidylinositol 3-kinase/protein kinase B signal transduction | -5.30 | 2.30 | 1.11 | 0.82 | 0.495 |
| **GO:0062197** | **cellular response to chemical stress** | -15.46 | 2.45 | 1.59 | 0.79 | 0.343 |
| GO:0032496 | response to lipopolysaccharide | 32496.00 | -8.38 | 2.50 | 1.78 | 0.813 |
| GO:0071396 | cellular response to lipid | 71396.00 | -11.25 | 2.76 | 3.19 | 0.782 |
| GO:0080134 | regulation of response to stress | -13.87 | 3.16 | 8.14 | 0.84 | 0.386 |
| **GO:0080135** | **regulation of cellular response to stress** | -9.88 | 2.73 | 3.01 | 0.84 | 0.329 |
| GO:0050727 | regulation of inflammatory response | 50727.00 | -5.76 | 2.63 | 2.38 | 0.845 |
| GO:0120254 | olefinic compound metabolic process | -5.48 | 2.20 | 0.88 | 0.98 | 0.110 |
| GO:1900407 | regulation of cellular response to oxidative stress | -6.25 | 1.34 | 0.12 | 0.88 | 0.450 |
| GO:1901652 | response to peptide | -11.52 | 2.94 | 4.87 | 0.82 | 0.410 |
| GO:1901653 | cellular response to peptide | -6.82 | 0.95 | 0.04 | 0.86 | 0.229 |
| GO:2000377 | regulation of reactive oxygen species metabolic process | -9.75 | 2.15 | 0.79 | 0.94 | 0.039 |
| GO:2000379 | positive regulation of reactive oxygen species metabolic process | -5.70 | 1.83 | 0.38 | 0.93 | 0.131 |

| TermID | Name | Value | LogSize | Frequency | Uniqueness | Dispensability |
|---|---|---|---|---|---|---|
| **GO:2001234** | **negative regulation of apoptotic signaling pathway** | -10.30 | 2.36 | 1.29 | 0.80 | 0.375 |
| GO:0043066 | negative regulation of apoptotic process | 43066.00 | -14.77 | 2.95 | 5.01 | 0.890 |
| GO:2001236 | regulation of extrinsic apoptotic signaling pathway | 2001236.00 | -6.24 | 2.20 | 0.89 | 0.808 |
| GO:2001243 | negative regulation of intrinsic apoptotic signaling pathway | 2001243.00 | -7.88 | 2.04 | 0.61 | 0.793 |

**Table S6** Redundancy-reduced GO Biological Process enrichment results for PTR-PD obtained using REVIGO semantic clustering. Legend: In highlighted REVIGO clusters, representative terms are shown in bold, whereas semantically related terms assigned to the same representative cluster are indicated using the same background color. TermID denotes the GO identifier, Name indicates the biological process, and Value corresponds to the input statistical value used for REVIGO analysis. LogSize and Frequency describe the relative size and occurrence frequency of each GO term in the Gene Ontology database, whereas Uniqueness and Dispensability indicate semantic distinctiveness and redundancy, respectively. Lower dispensability values indicate less redundant and more representative GO terms.

| TermID | Name | Value | LogSize | Frequency | Uniqueness | Dispensability |
|---|---|---|---|---|---|---|
| GO:0000165 | MAPK cascade | -8.98 | 2.35 | 1.26 | 0.87 | 0.473 |
| GO:0001819 | positive regulation of cytokine production | -6.43 | 2.70 | 2.81 | 0.89 | 0.430 |
| GO:0002376 | immune system process | -5.79 | 3.39 | 13.66 | 1.00 | 0.000 |
| GO:0002682 | regulation of immune system process | -8.90 | 3.22 | 9.29 | 0.96 | 0.048 |
| GO:0006629 | lipid metabolic process | -13.11 | 3.09 | 6.85 | 0.97 | 0.178 |
| **GO:0006631** | **fatty acid metabolic process** | -12.71 | 2.52 | 1.85 | 0.93 | 0.132 |
| GO:0006690 | icosanoid metabolic process | 6690.00 | -11.95 | 2.07 | 0.65 | 0.961 |
| GO:0008202 | steroid metabolic process | 8202.00 | -11.71 | 2.41 | 1.45 | 0.947 |
| GO:0006954 | inflammatory response | -7.04 | 2.78 | 3.37 | 0.90 | 0.127 |
| GO:0007169 | cell surface receptor protein tyrosine kinase signaling pathway | -13.90 | 2.65 | 2.48 | 0.87 | 0.121 |
| GO:0007611 | learning or memory | -6.07 | 2.46 | 1.61 | 1.00 | 0.000 |
| GO:0008210 | estrogen metabolic process | -7.87 | 1.60 | 0.22 | 0.93 | 0.494 |
| GO:0009410 | response to xenobiotic stimulus | -16.71 | 2.63 | 2.37 | 0.81 | 0.366 |
| GO:0009628 | response to abiotic stimulus | -15.85 | 3.06 | 6.41 | 0.91 | 0.141 |
| GO:0009636 | response to toxic substance | -11.61 | 2.41 | 1.43 | 0.82 | 0.341 |
| **GO:0009725** | **response to hormone** | -17.34 | 2.92 | 4.65 | 0.75 | 0.407 |
| GO:0032870 | cellular response to hormone stimulus | 32870.00 | -11.67 | 2.75 | 3.16 | 0.717 |
| GO:0048545 | response to steroid hormone | 48545.00 | -9.12 | 2.45 | 1.58 | 0.737 |
| GO:0070848 | response to growth factor | 70848.00 | -12.23 | 2.72 | 2.92 | 0.845 |
| GO:0010038 | response to metal ion | -10.89 | 2.54 | 1.95 | 0.82 | 0.356 |
| GO:0010506 | regulation of autophagy | -6.29 | 2.58 | 2.11 | 0.93 | 0.174 |
| GO:0010817 | regulation of hormone levels | -10.99 | 2.76 | 3.20 | 0.96 | 0.030 |
| GO:0012501 | programmed cell death | -7.97 | 3.05 | 6.34 | 1.00 | 0.004 |
| GO:0019216 | regulation of lipid metabolic process | -9.49 | 2.53 | 1.88 | 0.93 | 0.165 |
| GO:0030162 | regulation of proteolysis | -7.22 | 2.59 | 2.16 | 0.89 | 0.227 |

| TermID | Name | Value | LogSize | Frequency | Uniqueness | Dispensability |
|---|---|---|---|---|---|---|
| GO:0030522 | intracellular receptor signaling pathway | -11.41 | 2.31 | 1.15 | 0.87 | 0.226 |
| GO:0033993 | response to lipid | -23.79 | 2.95 | 4.99 | 0.79 | 0.000 |
| **GO:0034614** | **cellular response to reactive oxygen species** | -14.00 | 2.10 | 0.70 | 0.76 | 0.462 |
| GO:0042542 | response to hydrogen peroxide | 42542.00 | -7.84 | 2.00 | 0.56 | 0.800 |
| **GO:0043269** | **regulation of monoatomic ion transport** | -5.80 | 2.61 | 2.30 | 0.95 | 0.034 |
| GO:0034762 | regulation of transmembrane transport | 34762.00 | -5.79 | 2.60 | 2.25 | 0.949 |
| **GO:0043408** | **regulation of MAPK cascade** | -14.10 | 2.81 | 3.66 | 0.85 | 0.042 |
| GO:0032102 | negative regulation of response to external stimulus | 32102.00 | -7.12 | 2.60 | 2.25 | 0.877 |
| GO:0070372 | regulation of ERK1 and ERK2 cascade | 70372.00 | -7.13 | 2.45 | 1.57 | 0.868 |
| GO:1902532 | negative regulation of intracellular signal transduction | 1902532.00 | -12.75 | 2.88 | 4.22 | 0.836 |
| GO:1902533 | positive regulation of intracellular signal transduction | 1902533.00 | -14.39 | 3.05 | 6.35 | 0.826 |
| GO:0043525 | positive regulation of neuron apoptotic process | -5.70 | 1.82 | 0.37 | 0.92 | 0.485 |
| **GO:0043549** | **regulation of kinase activity** | -16.24 | 2.38 | 1.35 | 0.86 | 0.000 |
| GO:0033138 | positive regulation of peptidyl-serine phosphorylation | 33138.00 | -7.56 | 1.32 | 0.11 | 0.864 |
| GO:0043405 | regulation of MAP kinase activity | 43405.00 | -9.02 | 1.81 | 0.36 | 0.861 |
| GO:0045860 | positive regulation of protein kinase activity | 45860.00 | -10.60 | 2.07 | 0.66 | 0.836 |
| GO:0071900 | regulation of protein serine/threonine kinase activity | 71900.00 | -10.37 | 2.07 | 0.65 | 0.854 |
| GO:0045428 | regulation of nitric oxide biosynthetic process | -5.45 | 1.76 | 0.31 | 0.94 | 0.136 |
| **GO:0046777** | **protein autophosphorylation** | -14.07 | 2.06 | 0.65 | 0.94 | 0.098 |
| GO:0018105 | peptidyl-serine phosphorylation | 18105.00 | -13.92 | 1.59 | 0.21 | 0.944 |
| GO:0038083 | peptidyl-tyrosine autophosphorylation | 38083.00 | -7.21 | 0.95 | 0.04 | 0.949 |
| GO:0048511 | rhythmic process | -9.49 | 2.43 | 1.51 | 1.00 | 0.000 |
| GO:0050865 | regulation of cell activation | -6.88 | 2.81 | 3.62 | 0.95 | 0.048 |
| GO:0051897 | positive regulation of phosphatidylinositol 3-kinase/protein kinase B signal transduction | -6.73 | 2.30 | 1.11 | 0.86 | 0.495 |
| GO:0070482 | response to oxygen levels | -6.01 | 2.52 | 1.85 | 0.92 | 0.116 |
| GO:0071276 | cellular response to cadmium ion | -5.58 | 1.45 | 0.15 | 0.83 | 0.391 |
| **GO:0071396** | **cellular response to lipid** | -17.51 | 2.76 | 3.19 | 0.72 | 0.383 |
| GO:0032355 | response to estradiol | 32355.00 | -7.16 | 2.06 | 0.64 | 0.776 |
| GO:0032496 | response to lipopolysaccharide | 32496.00 | -11.13 | 2.50 | 1.78 | 0.752 |
| GO:0051384 | response to glucocorticoid | 51384.00 | -6.43 | 2.14 | 0.76 | 0.749 |
| GO:0062197 | cellular response to chemical stress | 62197.00 | -16.28 | 2.45 | 1.59 | 0.778 |

| TermID | Name | Value | LogSize | Frequency | Uniqueness | Dispensability |
|---|---|---|---|---|---|---|
| GO:0071383 | cellular response to steroid hormone stimulus | 71383.00 | -7.13 | 2.19 | 0.87 | 0.724 |
| GO:0071466 | cellular response to xenobiotic stimulus | -8.17 | 2.29 | 1.10 | 0.79 | 0.488 |
| **GO:0080135** | **regulation of cellular response to stress** | -10.32 | 2.73 | 3.01 | 0.87 | 0.329 |
| GO:0031347 | regulation of defense response | 31347.00 | -9.18 | 2.93 | 4.83 | 0.870 |
| GO:0098754 | detoxification | -6.80 | 2.18 | 0.84 | 0.83 | 0.318 |
| GO:0120254 | olefinic compound metabolic process | -16.15 | 2.20 | 0.88 | 0.98 | 0.000 |
| GO:1900407 | regulation of cellular response to oxidative stress | -7.58 | 1.34 | 0.12 | 0.91 | 0.450 |
| **GO:1901653** | **cellular response to peptide** | -9.60 | 0.95 | 0.04 | 0.84 | 0.231 |
| GO:0034097 | response to cytokine | 34097.00 | -5.39 | 2.93 | 4.77 | 0.787 |
| GO:1904645 | response to amyloid-beta | -5.97 | 1.74 | 0.30 | 0.83 | 0.281 |
| GO:2000377 | regulation of reactive oxygen species metabolic process | -10.53 | 2.15 | 0.79 | 0.94 | 0.149 |
| **GO:2001233** | **regulation of apoptotic signaling pathway** | -9.10 | 2.58 | 2.15 | 0.85 | 0.402 |
| GO:0043069 | negative regulation of programmed cell death | 43069.00 | -16.43 | 2.97 | 5.23 | 0.902 |
| GO:2001243 | negative regulation of intrinsic apoptotic signaling pathway | 2001243.00 | -6.88 | 2.04 | 0.61 | 0.842 |

**Table S7.** Redundancy-reduced GO Biological Process enrichment results for PTR-ALS obtained using REVIGO semantic clustering. Legend: In highlighted REVIGO clusters, representative terms are shown in bold, whereas semantically related terms assigned to the same representative cluster are indicated using the same background color. TermID denotes the GO identifier, Name indicates the biological process, and Value corresponds to the input statistical value used for REVIGO analysis. LogSize and Frequency describe the relative size and occurrence frequency of each GO term in the Gene Ontology database, whereas Uniqueness and Dispensability indicate semantic distinctiveness and redundancy, respectively. Lower dispensability values indicate less redundant and more representative GO terms.

| TermID | Name | Value | LogSize | Frequency | Uniqueness | Dispensability |
|---|---|---|---|---|---|---|
| GO:0000165 | MAPK cascade | -8.51 | 2.35 | 1.26 | 0.86 | 0.228 |
| GO:0001775 | cell activation | -6.22 | 2.89 | 4.35 | 0.98 | 0.176 |
| GO:0002682 | regulation of immune system process | -6.05 | 3.22 | 9.29 | 0.95 | 0.049 |
| GO:0006915 | apoptotic process | -9.60 | 3.03 | 6.06 | 1.00 | 0.005 |
| GO:0007169 | cell surface receptor protein tyrosine kinase signaling pathway | -14.34 | 2.65 | 2.48 | 0.86 | 0.164 |
| GO:0007399 | nervous system development | -6.08 | 3.36 | 12.96 | 0.98 | 0.247 |
| GO:0007611 | learning or memory | -7.42 | 2.46 | 1.61 | 0.98 | 0.000 |
| GO:0009410 | response to xenobiotic stimulus | -12.42 | 2.63 | 2.37 | 0.83 | 0.363 |
| GO:0009628 | response to abiotic stimulus | -15.36 | 3.06 | 6.41 | 0.91 | 0.118 |
| GO:0009636 | response to toxic substance | -8.27 | 2.41 | 1.43 | 0.84 | 0.295 |
| **GO:0009725** | **response to hormone** | -16.23 | 2.92 | 4.65 | 0.76 | 0.343 |
| GO:0032870 | cellular response to hormone stimulus | 32870.00 | -9.37 | 2.75 | 3.16 | 0.744 |
| GO:0048545 | response to steroid hormone | 48545.00 | -7.17 | 2.45 | 1.58 | 0.769 |
| GO:0070848 | response to growth factor | 70848.00 | -12.90 | 2.72 | 2.92 | 0.849 |
| GO:0010038 | response to metal ion | -10.58 | 2.54 | 1.95 | 0.83 | 0.353 |
| GO:0010506 | regulation of autophagy | -7.97 | 2.58 | 2.11 | 0.92 | 0.177 |
| GO:0030162 | regulation of proteolysis | -8.76 | 2.59 | 2.16 | 0.89 | 0.168 |
| GO:0030522 | intracellular receptor signaling pathway | -6.05 | 2.31 | 1.15 | 0.86 | 0.473 |
| GO:0031346 | positive regulation of cell projection organization | -6.20 | 2.55 | 1.99 | 0.94 | 0.160 |
| GO:0032496 | response to lipopolysaccharide | -7.59 | 2.50 | 1.78 | 0.79 | 0.481 |
| GO:0033554 | cellular response to stress | -13.95 | 3.22 | 9.34 | 0.87 | 0.395 |
| GO:0033993 | response to lipid | -16.21 | 2.95 | 4.99 | 0.81 | 0.407 |
| **GO:0042542** | **response to hydrogen peroxide** | -9.82 | 2.00 | 0.56 | 0.80 | 0.276 |
| GO:0070301 | cellular response to hydrogen peroxide | 70301.00 | -6.39 | 1.85 | 0.39 | 0.790 |

| TermID | Name | Value | LogSize | Frequency | Uniqueness | Dispensability |
| --- | --- | --- | --- | --- | --- | --- |
| **GO:0043549** | **regulation of kinase activity** | -14.22 | 2.38 | 1.35 | 0.87 | 0.000 |
| GO:0043406 | positive regulation of MAP kinase activity | 43406.00 | -5.82 | 1.57 | 0.20 | 0.709 |
| GO:0071900 | regulation of protein serine/threonine kinase activity | 71900.00 | -8.25 | 2.07 | 0.65 | 0.857 |
| GO:0045428 | regulation of nitric oxide biosynthetic process | -6.71 | 1.76 | 0.31 | 0.93 | 0.136 |
| GO:0050804 | modulation of chemical synaptic transmission | -5.87 | 2.71 | 2.88 | 0.85 | 0.383 |
| GO:0050865 | regulation of cell activation | -5.84 | 2.81 | 3.62 | 0.95 | 0.049 |
| GO:0062197 | cellular response to chemical stress | -18.24 | 2.45 | 1.59 | 0.80 | 0.000 |
| GO:0080134 | regulation of response to stress | -15.82 | 3.16 | 8.14 | 0.81 | 0.396 |
| **GO:0080135** | **regulation of cellular response to stress** | -10.73 | 2.73 | 3.01 | 0.81 | 0.336 |
| GO:0050727 | regulation of inflammatory response | 50727.00 | -5.87 | 2.63 | 2.38 | 0.814 |
| GO:1900407 | regulation of cellular response to oxidative stress | -6.88 | 1.34 | 0.12 | 0.86 | 0.450 |
| **GO:1902532** | **negative regulation of intracellular signal transduction** | -11.67 | 2.88 | 4.22 | 0.74 | 0.042 |
| GO:0043408 | regulation of MAPK cascade | 43408.00 | -11.30 | 2.81 | 3.66 | 0.757 |
| GO:0051896 | regulation of phosphatidylinositol 3-kinase/protein kinase B signal transduction | 51896.00 | -7.16 | 2.44 | 1.56 | 0.779 |
| GO:0051897 | positive regulation of phosphatidylinositol 3-kinase/protein kinase B signal transduction | 51897.00 | -7.03 | 2.30 | 1.11 | 0.771 |
| GO:0070372 | regulation of ERK1 and ERK2 cascade | 70372.00 | -6.05 | 2.45 | 1.57 | 0.769 |
| GO:2001236 | regulation of extrinsic apoptotic signaling pathway | 2001236.00 | -6.97 | 2.20 | 0.89 | 0.770 |
| GO:2001243 | negative regulation of intrinsic apoptotic signaling pathway | 2001243.00 | -7.43 | 2.04 | 0.61 | 0.750 |
| GO:1904645 | response to amyloid-beta | -5.92 | 1.74 | 0.30 | 0.84 | 0.404 |
| GO:2000377 | regulation of reactive oxygen species metabolic process | -10.73 | 2.15 | 0.79 | 0.93 | 0.149 |
| GO:2000379 | positive regulation of reactive oxygen species metabolic process | -7.51 | 1.83 | 0.38 | 0.92 | 0.138 |
| **GO:2001233** | **regulation of apoptotic signaling pathway** | -11.51 | 2.58 | 2.15 | 0.76 | 0.410 |
| GO:0043069 | negative regulation of programmed cell death | 43069.00 | -15.98 | 2.97 | 5.23 | 0.869 |

pharmaceuticals

MDPI

**Table S8.** Top representative GO Biological Process terms for PTR-AD selected after REVIGO semantic clustering. Representative terms within highlighted clusters are shown in bold, while semantically related terms assigned to the same representative cluster are marked with the same background color. The No. column indicates the ranked representative term or cluster, TermID denotes the GO identifier, and Name indicates the biological process.

| No. | TermID | Name |
|---|---|---|
| 1 | GO:0002376 | immune system process |
| 2 | GO:0007611 | learning or memory |
| 3 | GO:0008202 | steroid metabolic process |
| 4 | **GO:0009725** | **response to hormone** |
| | GO:0032870 | cellular response to hormone stimulus |
| | GO:0048545 | response to steroid hormone |
| | GO:0070848 | response to growth factor |
| 5 | **GO:0043408** | **regulation of MAPK cascade** |
| | GO:0070372 | regulation of ERK1 and ERK2 cascade |
| 6 | GO:0012501 | programmed cell death |
| 7 | GO:0019216 | regulation of lipid metabolic process |
| 8 | **GO:0034762** | **regulation of transmembrane transport** |
| | GO:0043269 | regulation of monoatomic ion transport |
| 9 | GO:0002682 | regulation of immune system process |
| 10 | GO:0050865 | regulation of cell activation |

**Table S9.** Top representative GO Biological Process terms for PTR-HD selected after REVIGO semantic clustering. Representative terms within highlighted clusters are shown in bold, while semantically related terms assigned to the same representative cluster are marked with the same background color. The No. column indicates the ranked representative term or cluster, TermID denotes the GO identifier, and Name indicates the biological process.

| No. | TermID | Name |
|---|---|---|
| 1 | GO:0002376 | immune system process |
| | **GO:0006631** | **fatty acid metabolic process** |
| 2 | GO:0006690 | icosanoid metabolic process |
| | GO:0008202 | steroid metabolic process |
| 3 | GO:0007611 | learning or memory |
| | **GO:0009725** | **response to hormone** |
| 4 | GO:0032870 | cellular response to hormone stimulus |
| | GO:0070848 | response to growth factor |
| 5 | **GO:0043408** | **regulation of MAPK cascade** |
| | GO:0051896 | regulation of phosphatidylinositol 3-kinase/protein kinase B signal transduction |
| 6 | GO:0012501 | programmed cell death |
| 7 | GO:0010817 | regulation of hormone levels |
| | **GO:0043269** | **regulation of monoatomic ion transport** |
| 8 | GO:0034762 | regulation of transmembrane transport |
| | GO:0051924 | regulation of calcium ion transport |
| 9 | GO:2000377 | regulation of reactive oxygen species metabolic process |
| 10 | GO:0002682 | regulation of immune system process |

**Table S10.** Top representative GO Biological Process terms for PTR-PD selected after REVIGO semantic clustering. Representative terms within highlighted clusters are shown in bold, while semantically related terms assigned to the same representative cluster are marked with the same background color. The No. column indicates the ranked representative term or cluster, TermID denotes the GO identifier, and Name indicates the biological process.

| No. | TermID | Name |
|---|---|---|
| 1 | GO:0002376 | immune system process |
| 2 | GO:0007611 | learning or memory |
| 3 | GO:0033993 | response to lipid |
| 4 | **GO:0043549** | **regulation of kinase activity** |
| | GO:0033138 | positive regulation of peptidyl-serine phosphorylation |
| | GO:0043405 | regulation of MAP kinase activity |
| | GO:0045860 | positive regulation of protein kinase activity |
| | GO:0071900 | regulation of protein serine/threonine kinase activity |
| 5 | GO:0048511 | rhythmic process |
| 6 | GO:0120254 | olefinic compound metabolic process |
| 7 | GO:0012501 | programmed cell death |
| 8 | GO:0010817 | regulation of hormone levels |
| 9 | **GO:0043269** | **regulation of monoatomic ion transport** |
| | GO:0034762 | regulation of transmembrane transport |
| 10 | **GO:0043408** | **regulation of MAPK cascade** |
| | GO:1902533 | positive regulation of intracellular signal transduction |
| | GO:1902532 | negative regulation of intracellular signal transduction |
| | GO:0070372 | regulation of ERK1 and ERK2 cascade |
| | GO:0032102 | negative regulation of response to external stimulus |

**Table S11.** Top representative GO Biological Process terms for PTR-ALS selected after REVIGO semantic clustering. Representative terms within highlighted clusters are shown in bold, while semantically related terms assigned to the same representative cluster are marked with the same background color. The No. column indicates the ranked representative term or cluster, TermID denotes the GO identifier, and Name indicates the biological process.

| No. | TermID | Name |
|---|---|---|
| 1 | GO:0007611 | learning or memory |
| 2 | **GO:0043549** | **regulation of kinase activity** |
| | GO:0043406 | positive regulation of MAP kinase activity |
| | GO:0071900 | regulation of protein serine/threonine kinase activity |
| 3 | GO:0062197 | cellular response to chemical stress |
| 4 | GO:0006915 | apoptotic process |
| 5 | **GO:1902532** | **negative regulation of intracellular signal transduction** |
| | GO:2001243 | negative regulation of intrinsic apoptotic signaling pathway |
| | GO:0043408 | regulation of MAPK cascade |
| | GO:0070372 | regulation of ERK1 and ERK2 cascade |
| | GO:2001236 | regulation of extrinsic apoptotic signaling pathway |
| | GO:0051897 | positive regulation of phosphatidylinositol 3-kinase/protein kinase B signal transduction |
| | GO:0051896 | regulation of phosphatidylinositol 3-kinase/protein kinase B signal transduction |
| 6 | GO:0050865 | regulation of cell activation |
| 7 | GO:0002682 | regulation of immune system process |
| 8 | GO:0009628 | response to abiotic stimulus |
| 9 | GO:0045428 | regulation of nitric oxide biosynthetic process |
| 10 | GO:2000379 | positive regulation of reactive oxygen species metabolic process |

**Table S12.** Hub network (Top 5% of degree value) and bottleneck network (Top 5% of betweenness value - BC) of pterostilbene-Alzheimer's disease PPI giant network. STRING protein identifiers (STRING name) were converted to canonical gene symbols (display name). Green-highlighted cells indicate proteins common to both the hub and bottleneck networks. Red-highlighted cells indicate proteins without connections, which were excluded from further analysis. All data presented in this table are available as an .xlsx file ('PTR-AD_top5%_Results_summary') in the online repository (https://doi.org/10.18150/HNUSRO, file: 'PPI network_results.zip').

| Top 5% of degree value | | | |
|---|---|---|---|
| STRING name | display name | degree | BC |
| 9606.ENSP00000362680 | SRC | 23 | 2038 |
| 9606.ENSP00000405330 | ESR1 | 22 | 4128 |
| 9606.ENSP00000335153 | HSP90AA1 | 21 | 3617 |
| 9606.ENSP00000263967 | PIK3CA | 20 | 1152 |
| 9606.ENSP00000309591 | PRKACA | 19 | 1170 |
| 9606.ENSP00000359719 | PRKACB | 18 | 696 |
| 9606.ENSP00000501150 | PIK3CB | 18 | 518 |
| 9606.ENSP00000215832 | MAPK1 | 16 | 1412 |
| 9606.ENSP00000378974 | MAPK8 | 16 | 1046 |
| 9606.ENSP00000394560 | MAPK9 | 15 | 373 |

| Top 5% of BC value | | | |
|---|---|---|---|
| STRING name | display name | degree | BC |
| 9606.ENSP00000405330 | ESR1 | 22 | 4128 |
| 9606.ENSP00000379683 | CYP19A1 | 7 | 4058 |
| 9606.ENSP00000335153 | HSP90AA1 | 21 | 3617 |
| 9606.ENSP00000498939 | CYP3A4 | 15 | 3070 |
| 9606.ENSP00000340820 | MAPT | 9 | 2055 |
| 9606.ENSP00000362680 | SRC | 23 | 2038 |
| 9606.ENSP00000378488 | CYP1A1 | 11 | 1881 |
| 9606.ENSP00000284981 | APP | 6 | 1735 |
| 9606.ENSP00000242057 | AHR | 3 | 1520 |
| 9606.ENSP00000245960 | CDC25B | 7 | 1491 |

**Table S13.** Hub network (Top 5% of degree value) and bottleneck network (Top 5% of betweenness value - BC) of pterostilbene-Huntington's disease PPI giant network. STRING protein identifiers (STRING name) were converted to canonical gene symbols (display name). Green-highlighted cells indicate proteins common to both the hub and bottleneck networks. All data presented in this table are available as an .xlsx file ('PTR-HD_top5%_Results_summary') in the online repository (https://doi.org/10.18150/HNUSRO, file: 'PPI network_results.zip').

| Top 5% of degree value | | | |
|---|---|---|---|
| STRING name | display name | degree | BC |
| 9606.ENSP00000362680 | SRC | 20 | 991 |
| 9606.ENSP00000405330 | ESR1 | 20 | 665 |
| 9606.ENSP00000335153 | HSP90AA1 | 19 | 1352 |
| 9606.ENSP00000309591 | PRKACA | 16 | 370 |
| 9606.ENSP00000215832 | MAPK1 | 16 | 605 |
| 9606.ENSP00000263967 | PIK3CA | 16 | 392 |
| 9606.ENSP00000359719 | PRKACB | 15 | 200 |

| Top 5% of BC value | | | |
|---|---|---|---|
| STRING name | display name | degree | BC |
| 9606.ENSP00000335153 | HSP90AA1 | 19 | 1352 |
| 9606.ENSP00000362680 | SRC | 20 | 991 |
| 9606.ENSP00000340820 | MAPT | 8 | 795 |
| 9606.ENSP00000284981 | APP | 5 | 688 |
| 9606.ENSP00000405330 | ESR1 | 20 | 665 |
| 9606.ENSP00000311032 | CASP3 | 9 | 623 |
| 9606.ENSP00000215832 | MAPK1 | 16 | 605 |

**Table S14**. Hub network (Top 5% of degree value) and bottleneck network (Top 5% of betweenness value - BC) of pterostilbene-Parkinson's disease PPI giant network. STRING protein identifiers (STRING name) were converted to canonical gene symbols (display name). Green-highlighted cells indicate proteins common to both the hub and bottleneck networks. All data presented in this table are available as an .xlsx file ('PTR-PD_top5%_Results_summary') in the online repository (https://doi.org/10.18150/HNUSRO, file: 'PPI network_results.zip').

Top 5% of degree value

| STRING name | display name | degree | BC |
|---|---|---|---|
| 9606.ENSP00000362680 | SRC | 22 | 1754 |
| 9606.ENSP00000335153 | HSP90AA1 | 20 | 3152 |
| 9606.ENSP00000405330 | ESR1 | 20 | 3644 |
| 9606.ENSP00000263967 | PIK3CA | 18 | 828 |
| 9606.ENSP00000309591 | PRKACA | 18 | 914 |
| 9606.ENSP00000359719 | PRKACB | 17 | 588 |
| 9606.ENSP00000215832 | MAPK1 | 16 | 1254 |
| 9606.ENSP00000501150 | PIK3CB | 16 | 289 |
| 9606.ENSP00000498939 | CYP3A4 | 15 | 2585 |

Top 5% of BC value

| STRING name | display name | degree | BC |
|---|---|---|---|
| 9606.ENSP00000379683 | CYP19A1 | 7 | 3750 |
| 9606.ENSP00000405330 | ESR1 | 20 | 3644 |
| 9606.ENSP00000335153 | HSP90AA1 | 20 | 3152 |
| 9606.ENSP00000498939 | CYP3A4 | 15 | 2585 |
| 9606.ENSP00000362680 | SRC | 22 | 1754 |
| 9606.ENSP00000378488 | CYP1A1 | 11 | 1585 |
| 9606.ENSP00000340820 | MAPT | 9 | 1571 |
| 9606.ENSP00000343925 | ESR2 | 11 | 1326 |
| 9606.ENSP00000242057 | AHR | 3 | 1297 |

**Table S15.** Hub network (Top 5% of degree value) and bottleneck network (Top 5% of betweenness value - BC) of pterostilbene-amyotrophic lateral sclerosis PPI giant network. STRING protein identifiers (STRING name) were converted to canonical gene symbols (display name). Green-highlighted cells indicate proteins common to both the hub and bottleneck networks. All data presented in this table are available as an .xlsx file ('PTR-ALS_top5%_Results_summary') in the online repository (https://doi.org/10.18150/HNUSRO, file: 'PPI network_results.zip').

| Top 5% of degree value | | | |
|---|---|---|---|
| STRING name | display name | degree | BC |
| 9606.ENSP00000362680 | SRC | 21 | 722 |
| 9606.ENSP00000335153 | HSP90AA1 | 19 | 963 |
| 9606.ENSP00000263967 | PIK3CA | 18 | 272 |
| 9606.ENSP00000405330 | ESR1 | 17 | 340 |
| 9606.ENSP00000501150 | PIK3CB | 16 | 157 |
| 9606.ENSP00000309591 | PRKACA | 14 | 220 |

| Top 5% of BC value | | | |
|---|---|---|---|
| STRING name | display name | BC | degree |
| 9606.ENSP00000335153 | HSP90AA1 | 963 | 19 |
| 9606.ENSP00000362680 | SRC | 722 | 21 |
| 9606.ENSP00000340820 | MAPT | 609 | 8 |
| 9606.ENSP00000311032 | CASP3 | 485 | 9 |
| 9606.ENSP00000284981 | APP | 470 | 5 |
| 9606.ENSP00000324806 | GSK3B | 421 | 10 |

**Table S16.** Average and standard deviation of the minimum distance factor analysis between the ESR1, HSP90, and SCR binding pocket residues and pterostilbene (PTR) for the initial docking poses across 3 replicas.

| Systems | Rep-1 | Rep-2 | Rep-3 |
|---|---|---|---|
| ESR1-PTR | 0.6787 ± 0.0545 | 0.6778 ± 0.0468 | 0.7387 ± 0.1043 |
| HSP90-PTR | 0.8768 ± 0.0554 | 0.8519 ± 0.0475 | 0.8497 ± 0.0481 |
| SCR-PTR | 4.6572 ± 1.5285 | 3.8476 ± 2.2325 | 4.9742 ± 1.6076 |

**Table S17.** Average and standard deviation of the MM/GBSA between the ESR1 and HSP90 proteins and pterostilbene (PTR) across 3 replicas.

| Systems | Rep-1 | Rep-2 | Rep-3 |
|---|---|---|---|
| ESR1-PTR | -35.14 ± 0.94 | -31.77 ± 1.63 | -28.18 ± 0.95 |
| HSP90-PTR | -23.80 ± 0.92 | -21.23 ± 0.63 | -20.22 ± 1.29 |

**Table S18.** Threshold-sensitivity check showing the number of overlapping PTR–disease targets identified using different GeneCards relevance-score cutoffs (>5, >10, and >20). The underlying data are provided in the RepOD repository (https://doi.org/10.18150/HNUSRO) in the file "Threshold-sensitivity check GeneCards.tab".

| Relevance score | >5 | >10 | >20 |
|---|---|---|---|
| PTR-AD | n=181 | n=146 | n=87 |
| PTR-HD | n=128 | n=105 | n=56 |
| PTR-PD | n=165 | n=139 | n=81 |
| PTR-ALS | n=109 | n=76 | n=33 |
